# Diet of a rare herbivore based on DNA metabarcoding of feces: Selection, seasonality, and survival

**DOI:** 10.1002/ece3.6488

**Published:** 2020-06-30

**Authors:** Amanda R. Goldberg, Courtney J. Conway, David C. Tank, Kimberly R. Andrews, Digpal S. Gour, Lisette P. Waits

**Affiliations:** ^1^ Idaho Cooperative Fish and Wildlife Research Unit Department of Fish and Wildlife Sciences University of Idaho Moscow ID USA; ^2^ U.S. Geological Survey Idaho Cooperative Fish and Wildlife Research Unit University of Idaho Moscow ID USA; ^3^ Department of Biological Sciences and Stillinger Herbarium University of Idaho Moscow ID USA; ^4^ Department of Fish & Wildlife Sciences and Institute for Bioinformatics and Evolutionary Studies (IBEST) University of Idaho Moscow ID USA; ^5^ Department of Fish and Wildlife Sciences University of Idaho Moscow ID USA

**Keywords:** diet composition, fitness consequences, ground squirrel, noninvasive diet analysis, phenology, *Urocitellus brunneus*

## Abstract

In herbivores, survival and reproduction are influenced by quality and quantity of forage, and hence, diet and foraging behavior are the foundation of an herbivore's life history strategy. Given the importance of diet to most herbivores, it is imperative that we know the species of plants they prefer, especially for herbivorous species that are at risk for extinction. However, it is often difficult to identify the diet of small herbivores because: (a) They are difficult to observe, (b) collecting stomach contents requires sacrificing animals, and (c) microhistology requires accurately identifying taxa from partially digested plant fragments and likely overemphasizes less‐digestible taxa. The northern Idaho ground squirrel (*Urocitellus brunneus*) is federally threatened in the United States under the Endangered Species Act. We used DNA metabarcoding techniques to identify the diet of 188 squirrels at 11 study sites from fecal samples. We identified 42 families, 126 genera, and 120 species of plants in the squirrel's diet. Our use of three gene regions was beneficial because reliance on only one gene region (e.g., only *trnL*) would have caused us to miss >30% of the taxa in their diet. Northern Idaho ground squirrel diet differed between spring and summer, frequency of many plants in the diet differed from their frequency within their foraging areas (evidence of selective foraging), and several plant genera in their diet were associated with survival. Our results suggest that while these squirrels are generalists (they consume a wide variety of plant species), they are also selective and do not eat plants relative to availability. Consumption of particular genera such as *Perideridia* may be associated with higher overwinter survival.

## Graphical abstract

Diet and foraging behavior are the foundation of an herbivore's life history strategy and it is imperative that we know the species of plants they prefer. We used DNA metabarcoding techniques to noninvasively sample the threatened northern Idaho ground squirrel (*Urocitellus brunneus*) diet. We used three gene regions and found that diets differed on the genus level between season, availability, and survival was associated with particular plants.

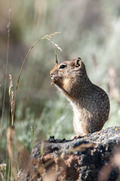

## 1 INTRODUCTION

You are what you eat. There is a lot of truth in this old French aphorism because animals are often defined by their diet and the search for food typically affects most other aspects of an animal's behavior, ecology, and survival. For example, herbivores are often thought to be food limited (Belovsky, 1986; Bobek, 1977; Fryxell, 1987; Sinclair, Dublin, & Borner, 1985; Skogland, 1985; White, 2008) and reductions in high‐quality forage may reduce population size via impacts to demographic parameters. Detailed knowledge of diet breadth and preferred food items is important for exploring optimal foraging theory which assumes that the main goal of a generalist herbivore is to maximize quantity or quality of food while foraging, or to obtain food efficiently while avoiding predation (Belovsky, 1986; Charnov, 1976; MacArthur & Pianka, 1966). Both survival and reproduction of herbivores are associated with increased energy intake (Ritchie, 1990; White, 1983). Hence, foraging behaviors that obtain a sufficient amount of high‐quality forage quickly and safely are assumed to be under strong selection for many herbivores, and diet and foraging behavior are the foundation of an herbivore's life history strategy. Herbivores face a number of anthropogenic threats that may reduce food availability or food quality such as: (a) changes in plant community composition due to invasive species (D’Antonio and Vitousek 1992, Drake et al., 2016; Freeman et al., 2014), (b) reduced plant biomass due to livestock grazing (Hayes & Holl, 2003), and (c) climate‐induced reductions in optimal forage plants due to changes in precipitation and temperature (Bertrand et al., 2011; Thuiller, Lavorel, Araújo, Sykes, & Prentice, 2005). Thus, a reduction in the availability of high‐quality food will likely lead to either longer foraging times (and hence high predation risk) or poor body condition (and lower reproduction and survival). Hence, it is imperative that we understand both diet breadth and optimal food items in the diet (e.g., use versus availability) to better manage herbivorous mammals of conservation concern.

Most mammalian herbivores consume many species of plants as a way to consume small doses of a diverse array of plant secondary compounds (Torregrossa, Azzara, & Dearing, 2011). Furthermore, mammals with diverse diets may exhibit preferences for specific plants because nutrients vary among food items (e.g., protein content, or fiber; Randolph & Cameron, 2001). Generalist herbivores eat many different plants and/or components of plants (i.e., seeds, roots, leaves; Eshelman & Jenkins, 1989), but they do not necessarily eat everything they come into contact with and in proportion to availability (Rogers & Gano, 1980). Small mammals may be particularly likely to select specific plants because they have short guts and hence can only eat so much food at a time. Hence, small herbivores often face foraging trade‐offs between handling plant defenses, minimizing search time, and maximizing nutritional requirements per unit consumed (Belovsky, 1986). Previous studies have found that diets (on the scale of growth form classification; grasses, shrubs, forbs) impacted reproduction (Ritchie, 1990) and diet preference changed by season and drought (Van Horne, Schooley, & Sharpe, 1998). Furthermore, these trade‐offs likely change seasonally as plant defenses, food availability, and nutritional requirements change.

Animals that hibernate may be particularly sensitive to changes in food quantity or quality because they may have different nutritional needs than nonhibernators and these nutrients must be obtained during a relatively short period of time each year. Many hibernating ground squirrels alter their diets seasonally, and often switch to eating plants high in particular polyunsaturated fatty acids (e.g., seeds) prior to hibernation (Frank, 1994; Lehmer, Biggins, & Antolin, 2006) which lead to increased overwinter survival (Ruf & Arnold, 2008). Therefore, documenting seasonal changes in preferred forage is particularly important for hibernating herbivores, and diet studies need to have sufficient taxonomic resolution to detect species‐level shifts in preferred forage items.

Furthermore, diets may differ demographically due to different nutritional requirements. For instance, females may require additional nutrients during pregnancy and lactation which are energetically consuming (Barboza & Bowyer, 2000; Randolph & Cameron, 2001; Rothman, Dierenfeld, Hintz, & Pell, 2008). Juveniles may have different diets than adults for a number of reasons: (a) juveniles may need to forage close to their natal burrow (Van Horne et al., 1998), (b) juveniles may be less tolerant of secondary compounds and other plant chemical responses (Van Horne et al., 1998), and/or (c) juveniles have smaller guts and thus may need to adjust their diet accordingly (Demment & Van Soest, 1985).

The northern Idaho ground squirrel (*Urocitellus brunneus*) is a rare, federally threatened species that hibernates for approximately eight months each year (U.S. Fish and Wildlife Service 2003). Habitat loss is thought to be the cause of their past population declines and range contraction. Years of fire suppression have enabled conifer trees to encroach into meadows and forest openings where these ground squirrels live. Changes in canopy cover and loss of fire may have led to changes in food quantity or quality (Suronen & Newingham, 2013a). We need more detailed baseline information on the squirrels’ diet to better assess how management treatments affect preferred forage plants and how best to manage squirrel habitat in the future.

Northern Idaho ground squirrels have a diverse diet (Dyni & Yensen, 1996; Yensen, Shock, Tarifa, & Evans Mack, 2018), and thus are considered to be generalist foragers. However, previous studies focused on only seven sites out of ~119 known extant locations and five of the seven sites are within 5km and 45m elevation of one another. Furthermore, all previous noninvasive diet studies used microhistological techniques. However, new DNA metabarcoding techniques might provide higher resolution compared to microhistological methods (Bybee et al., 2011; Valentini et al., 2009). DNA metabarcoding may enable us to evaluate diets at more precise taxonomic levels and with less bias, and thereby may help better assess dietary preferences and identify forage items that impact survival.

**Figure 1:**
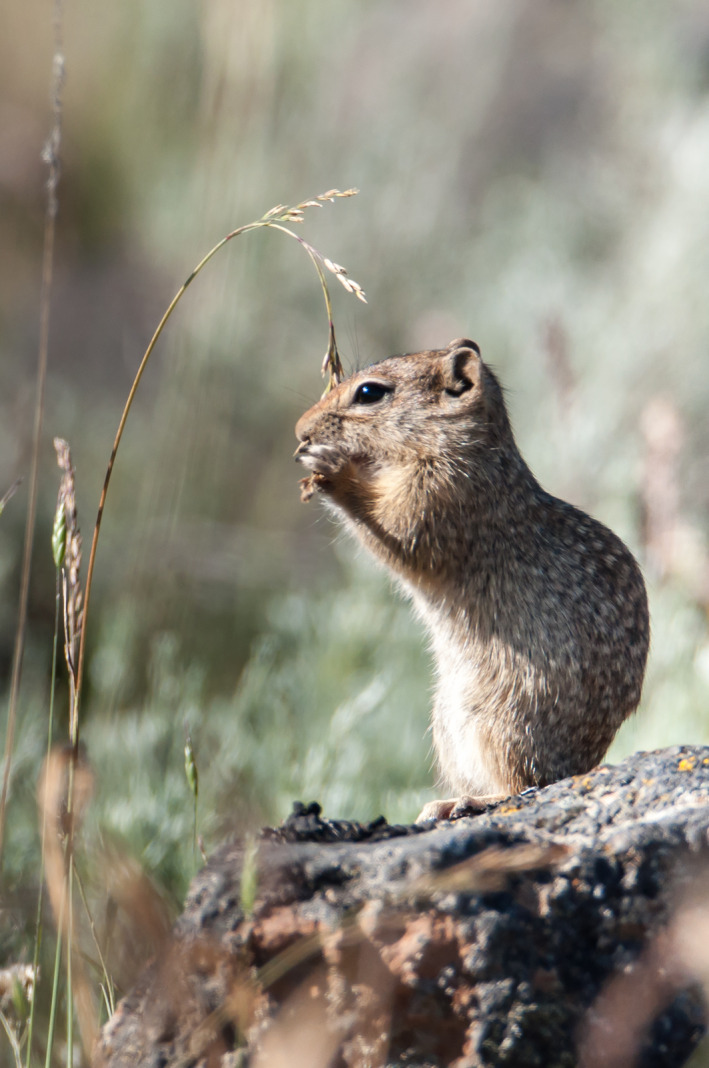
Cover Image: A foraging northern Idaho ground squirrel (*Urocitellus brunneus*)

To better understand diet breadth and optimal forage for this rare species, we addressed the following questions: (a) What plants are these squirrels consuming?, (b) Do they select for specific plants/nutrients compared to what is available?, (c) Do their diets differ by season? And, if so, do they differ because availability differs or because nutritional needs differ?, (d) Do diets differ between sexes due to differences in energetic needs from reproduction?, (e) Do diets differ between age classes?, and (f) Does diet impact overwinter survival during hibernation? Previous microhistological studies on ground squirrels have reported no difference in diet between males and females (Van Horne et al., 1998; Yensen et al., 2018). However, subtle differences between species, sexes, or age classes may be difficult to detect with microhistology (King & Schoenecker, 2019; Soininen et al., 2009). Understanding if and how diet varies seasonally and among sex and age classes would help determine which plants influence persistence of northern Idaho ground squirrel populations (Figure 1).

We used DNA metabarcoding of fecal pellets to identify both the composition and frequency of plants in the diet of northern Idaho ground squirrels. To address these needs, we designed studies to pursue five objectives:

1. Better document the diet of northern Idaho ground squirrels across a broader range of their distribution. Document both the fine scale (lowest taxonomic level we can for detailed analyses and understanding of their diet needs) and broad scale (growth form level so we can easily compare diet choices and needs to other studies and understand more general dietary needs).
2. Determine whether diet of northern Idaho ground squirrels differs among demographics. We tested the hypothesis that diets will differ between both age and sex classes.
3. Determine whether diet of northern Idaho ground squirrels differs by season. We tested the hypothesis that squirrels will consume different plants in the spring than the summer.
4. Determine whether any plants are preferred by northern Idaho ground squirrels (do northern Idaho ground squirrel select food items relative to their availability on the landscape). We tested the hypothesis that northern Idaho ground squirrels select particular plants (i.e., some plants are more common in squirrel's diet than would be predicted based on availability).
5. Determine whether particular plant genera are associated with squirrel survival. We tested the hypothesis that squirrels will select particular plants in the summer which will lead to increased overwinter survival.

## 2 MATERIALS AND METHODS

### 2.1 Study area and species

**Figure 2:**
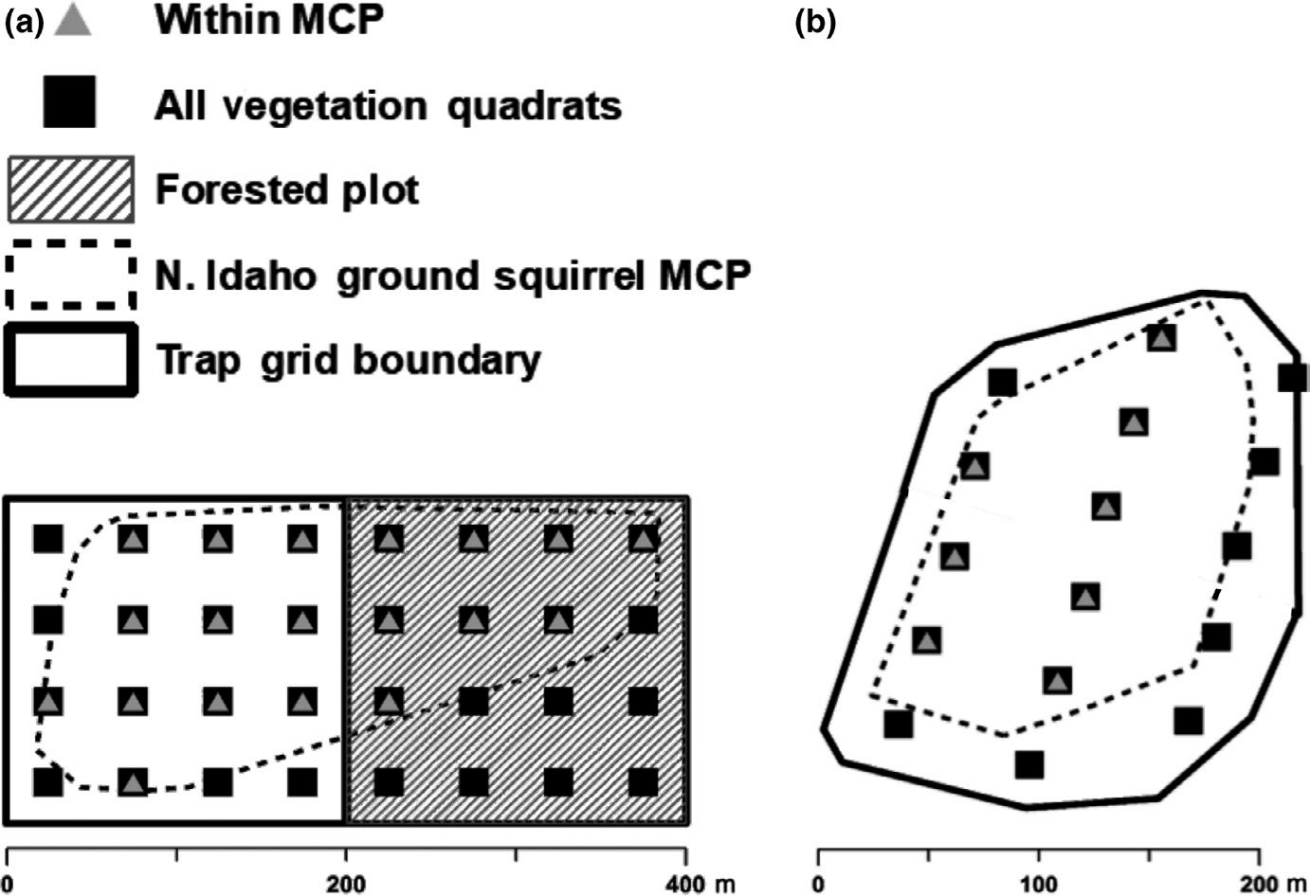
The two northern Idaho ground squirrel study site designs: (a) nine of the 13 study sites included two 4‐ha plots (one in nonforest and one in the adjacent forest) and (b) four of the 13 study sites included a 4–8‐ha plot in a nonforested area. Small black squares are the evenly spaced vegetation quadrats. We placed 16 quadrats in every 4‐ha plot. The dotted line represents the area occupied (minimum convex polygon) by northern Idaho ground squirrels at the site (based on trapping data). Gray triangles indicate the vegetation quadrats that are within the squirrel MCP

We collected northern Idaho ground squirrel fecal pellets in 2015 and 2016 from 13 study sites in Adams County, Idaho. The study sites were mostly in remote areas on lands managed by the U.S. Forest Service, Idaho Department of Lands, and privately owned lands. Study sites varied in elevation (1,280–1,700 m) and distance to nearest incorporated town (8–35 km straight line distance). The study sites included 13 of the 124 extant sites known to support northern Idaho ground squirrels, and were part of a long‐term restoration project employing two study designs: (a) 8‐ha plots that straddled the ecotone between forest and nonforest (meadow/clearing) (Figure 2a), and (b) 4‐ to 8‐ha nonforested plots (Figure 2b). Northern Idaho ground squirrels mate in the spring soon after females emerge from hibernacula in late March–April, reproduce only once per year, and immerge back into hibernacula in July–August (Yensen & Sherman 1997). Hence, squirrels should forage efficiently on the most preferred food items available to them because they have a short active season above ground (~4 months) when they must reproduce, raise offspring, and increase their body mass before reentering into hibernation.

### 2.2 Vegetation sampling

We used 1‐m^2^ quadrats to sample vegetation composition at all 13 study sites in Jun–Jul of 2015 and 2016. We placed quadrats equidistantly spaced throughout the study sites (four quadrats per 1‐ha; Figure 2). We recorded all plants present within each quadrat below waist level and identified all plants to the lowest taxonomic level possible. We examined two subsets of the vegetation data: (a) data from all of the 383 quadrats at all of the 13 study sites (16–32 quadrats per site; 4 quadrats/ha), hereafter referred to as vegetation quadrats, and (b) only data from the 191 quadrats that fell within the minimum convex polygons (MCPs), based on all trapped northern Idaho ground squirrels at each site, hereafter referred to as MCP vegetation quadrats (Figure 2). MCPs were calculated around all trap locations where we captured a squirrel within a site. We used the minimum bounding geometry – convex hull tool in ArcGIS 10.4.1 (Esri Inc) to calculate the MCPs. This approach allowed us to more rigorously address objective #4 above and to examine use versus availability at two nested spatial scales (throughout 4‐ha plots and only within MCPs used by squirrels).

### 2.3 Trapping methods

We trapped squirrels for two sessions per year at each site: in the spring (late Apr – early Jun) and in the summer (June–late July). We trapped squirrels for three or four nonconsecutive days within each session at each site as required by our permits. Each trap session lasted between nine and 21 days (mean = 15.6 ± 0.36 SE). Traps were checked continuously throughout the day approximately every 15–30 min. The number of hours we trapped each site per day varied based on weather (temperature and precipitation) and to conform to a coexisting study. During the spring trapping session, we started trapping ~30 min after dawn and stopped just before dusk (whenever above ground squirrel activity ceased) at nine sites and stopped at 1:00 p.m. at the four remaining sites. Summer trapping days were shorter because we ceased trapping if the ambient temperature rose above 27°C (to minimize risk to the squirrels whom are temperature sensitive). We used Tomahawk live traps (Tomahawk Live Trap Co; 13 × 13 × 41 cm and 15 × 15 × 50 cm) or focal traps we built to trap northern Idaho ground squirrels within a 4–8 ha trapping area. All Tomahawk traps were baited with a mixture of oats, peanut butter, and imitation vanilla extract.

We carried all squirrels that were trapped for the first time within each session to a processing station. We placed each trap with a captured squirrel on top of a paper towel for ~30 min. We placed all fecal pellets that fell onto the paper towel into a coin envelope. We placed the envelopes in a zip‐top bag with silicone gel beads to keep samples dry, and then placed them in a freezer at the end of the summer field season to minimize degradation. We used either 2 metal ear tags (National Band and Tag Co; model 1005‐1) or Passive Integrated Transponder (PIT) tags (Biomark Inc; model HPT12) to individually mark new captures. We visually identified both the sex and age (either juvenile or adult based on size and time of year) of all trapped individuals. We rarely captured juveniles in the spring, because they do not emerge from their natal burrows until the end of the spring trapping season (late May or early June). All individuals were returned to the exact location they were trapped after they were processed and any fecal samples they produced were collected. This study was performed under the auspices of University of Idaho IACUC protocol #2015‐53.

### 2.4 DNA extraction

We used QIAamp DNA Stool Mini Kit (Qiagen, Inc) to extract genomic DNA from the pellet samples in a laboratory dedicated to low quality DNA samples where no PCR products or concentrated DNA was present. One negative control was included in each extraction set of 20 samples to test for contamination. We only analyzed samples for which we had two or more pellets (between two and eight pellets with an average of four pellets). We combined the pellets from each sample together for DNA extraction because initial tests indicated that one pellet had low PCR success. Each individual pellet averaged 0.03 g (ranged from 0.01 g to 0.05 g). We analyzed 188 fecal samples from our 13 study sites (Table 1).

**Table 1:** Fecal samples collected in 2015 and 2016 from northern Idaho ground squirrels in Adams County, Idaho. Samples were collected from 13 different sites

Age	Spring			Summer			Total
	Female	Male	All	Female	Male	All	
Adult	41	22	63	32	21	53	116
Juvenile	1		1	44	27	71	72
Total	42	22	64	76	48	124	188

### 2.5 PCR and amplicon sequencing

We used three metabarcoding primer sets to amplify three gene regions: two fragments from the nuclear ribosomal (nr) DNA internal transcribed spacer (ITS) region, and a portion of the chloroplast (cp) DNA *trnL* intron (*trnL*). The two ITS fragments (ITS1 and ITS2; each ~200–300 bp) were amplified using the “universal” plant primer pairs its5/its2 (ITS1) and its3/its4 (ITS2) (Baldwin, 1992). To amplify a portion of the cpDNA *trnL* intron, a region of the plastome commonly sequenced for species‐level plant systematic studies, we designed universal primers for seed plants to amplify an ~200 bp portion of the 5′ end of the *trnL* intron (trnLi_SP_9F: TGGATTGAGCCTTGGTATGGAA, trnLi_SP_189R: AGCTTCCATTGAGTCTCTGCA), based on prior knowledge of the plant composition data that we collected as part of a companion study (Andrews et al. 2017). We downloaded sequences for the *trnL* intron region from GenBank for all species of seed plants known to occur at the study sites with available sequence data (132 species), sequences were aligned using MAFFT v.7.402 (Katoh & Standley, 2013). Primers were designed using Primer3 (Untergasser et al., 2012) for targets of 150–200 bp using default parameters except annealing temperature was set to 60°C (±2°C). No primer sequences with more than 3 repeated nucleotides of the same base (like AAAA) were allowed (Max Poly‐X = 3). To confirm the performance of these primers before using them for fecal samples, we initially conducted PCRs for each primer set on genomic DNA extracted from herbarium specimens of two plant species that occur in northern Idaho ground squirrel habitat: *Balsamorhiza sagittata* and *Salix scouleriana*.

PCR amplification followed a two‐round PCR strategy (including one PCR negative in each round). Following Uribe‐Convers, Settles, and Tank (2016), each target‐specific primer sequence contained a conserved sequence tag that was added to the 5' end at the time of oligonucleotide synthesis (CS1 for forward primers and CS2 for reverse primers). The purpose of the added CS1 and CS2 tails is to provide an annealing site for the second pair of primers. After an initial round of PCR using the CS‐tagged target‐specific primers (PCR1), a second round of PCR was used to add 8 bp sample‐specific barcodes and high‐throughput sequencing adapters to both the 5′ and 3′ ends of each PCR amplicon (PCR2), with a different barcode at each end of the amplicon (a “dual‐barcode”). From 5′ to 3′, the PCR2 primers included the reverse complement of the conserved sequence tags, sample‐specific 8 bp barcodes, and either Illumina P5 (CS1‐tagged forward primers) or P7 (CS2‐tagged reverse primers) sequencing adapters. Sequences for the CS1 and CS2 conserved sequence tags, barcodes, and sequencing adapters were taken from Uribe‐Convers et al. (2016). PCR1 (25 μl) reactions included 2.5 μl of 10× PCR buffer, 3 μl of 25 mM MgCl_2_, 0.30 μl of 20 mg/ml BSA, 1 μl of 10 mM dNTP mix, 0.125 μl of 10 μM CS1‐tagged target‐specific forward primer, 0.125 μl of 10 uM CS2‐tagged target‐specific reverse primer, 0.125 μl of 5,000 U/ml Taq DNA polymerase, 1 μl template of DNA, and PCR‐grade H_2_O to volume. The PCR1 cycling conditions included 95°C for 2 min., 20 cycles of 95°C for 2 min., 50–60°C for 1 min. (depending on *T*_m_ of target‐specific primers), 68°C for 1 min., and a final extension of 68°C for 10 min. PCR2 (20 μl) reactions included 2 μl of 10× PCR buffer, 3.6 μl of 25 mM MgCl_2_, 0.60 μl of 20 mg/ml BSA, 0.40 μl of 10 mM dNTP mix, 0.75 μl of 2 μM barcoded primer mix, 0.125 μl of 5,000 U/ml Taq DNA polymerase, 1 μl of PCR1 product as template, and PCR‐grade H_2_O to volume. The PCR2 cycling conditions included 95°C for 1 min., 15 cycles of 95°C for 30 s, 60°C for 30 s, 68°C for 1 min., and a final extension of 68°C for 5 min. Following PCR2, the resulting amplicons for the two ITS markers and the *trnL* marker were pooled together at approximately equimolar concentrations and sequenced on an Illumina MiSeq platform with 150 bp paired‐end reads using the 300 cycle MiSeq Sequencing v2 Micro kit on one‐sixth of a lane. The *trnL* marker was sequenced separately because it is a shorter amplicon.

### 2.6 Sequence processing

Pooled reads from the Illumina MiSeq run were demultiplexed using the dbcAmplicons pipeline (https://github.com/msettles/dbcAmplicons) following the workflow detailed in Uribe‐Convers et al. (2016). Sample‐specific dual‐barcodes and target‐specific primers were identified for each read pair and removed, allowing the default matching error of one base per barcode and four bases. Each read was annotated to include the sample name (based on the barcode sequences) and gene region (based on the primer sequences). To eliminate fungal contamination that may have been amplified with ITS and nonspecific amplification of poor PCR products for both gene regions, each read was screened against a user‐defined reference file of annotated sequences retrieved from GenBank (using the “‐screen” option in dbcAmplicons). Reads that mapped with default sensitivity settings were kept, and unique sequences were identified using the clustering approaches implemented in PURC v.1.02 (Rothfels, Pryer, & Li, 2017). The fluidigm2purc pipeline (Blischak et al., 2018) was used to convert demultiplexed data from dbcAmplicons to inputs for PURC. The fluidigm2purc pipeline takes the paired‐end FASTQ files and filters sequence reads using Sickle (Joshi & Fass, 2011); minimum length = 100 bp, PHRED threshold = 20). Fluidigm2purc merges the filtered paired‐end reads using FLASH2 (Magoč & Salzberg, 2011), and converts the resulting FASTQ files into FASTA files for each gene region with sequence header information that is compatible with the *purc_recluster.py* script (Rothfels et al., 2017). The *purc_recluster.py* script was used to iteratively run chimera detection and sequence clustering (performed with USEARCH; Edgar, 2010; Edgar, Haas, Clemente, Quince, & Knight, 2011)) on each gene region individually to produce a reduced set of putative haplotypes that includes information about the number of original reads forming each cluster. We used the iterative clustering thresholds of 0.975, 0.950, 0.925, and 0.975, and defaults for other settings (for more details on fluidigm2purc and PURC see https://github.com/pblischak/fluidigm2purc and https://bitbucket.org/crothfels/purc, respectively).

### 2.7 Sequence identification

Clustered sequences recovered from PURC were identified using a combination of GenBank blast hits, and an annotated list of plants known to occur at the 13 study sites based on species documented within 383 1‐m^2^ quadrats that we sampled at each of the 13 study sites (see below), and information from previous studies at these sites (Dyni & Yensen, 1996; Suronen & Newingham, 2013b; Yensen, Teresa, Evans Mack, Wagner, & Shock, 2013). To identify the closest sequence in GenBank (accessed January 18, 2018), we used blastn v.2.60 (Altschul, Gish, Miller, Myers, & Lipman, 1990) from the command line ncbi tools and recorded the top hit based on comparison with the annotated list of plants known to occur at all sites combined.

### 2.8 Fecal sample diet identification

We created a master list of all known plants that we detected from at least one of our 13 study sites. In addition, we added two families, 20 genera, and 83 species that we did not record on our 383 vegetation quadrats, but were identified in past studies from at least one of our 13 study sites (Dyni & Yensen, 1996; Suronen & Newingham, 2013b; Yensen et al., 2013). Combined, we had a list of 276 species, 188 genera, and 44 families on the master list that we believe represented a nearly complete list of the possible food plants that were available to northern Idaho ground squirrels at our 13 study sites (Table S1). We compared the lowest taxonomic level from the top GenBank blast hits to our list of 276 plants (e.g., if the top hit was *Lupinus laxiflorus* but we only identified *Lupinus* in the field to genus, then we assigned all top blast hits of *Lupinus laxiflorus* to *Lupinus* spp.). In some cases, we were able to deduce a lower taxonomic level assignment than was identified on GenBank if only one known plant occurred in that category under the next higher taxonomic level (e.g., only one species within the genus *Microseris* is on our master list of 276 species, so any top hit of *Microseris* spp. was assumed to be *Microseris nutans*). We used this information to create a list of species identified in the diet based on genetic data informed by vegetation assessments (Table S1). However, for all subsequent analyses, we used genus level assignments. Genus was the lowest taxonomic resolution possible for many plants by field technicians conducting our vegetation quadrats.

### 2.9 Data analysis

#### 2.9.1 Initial evaluation of the diet data

We used the iNEXT R package to compile sample‐based rarefaction curves for our fecal data (Chao et al., 2014, Hsieh et al. 2018) to evaluate whether we collected enough fecal pellet samples to document the breadth of the squirrels diet (i.e., did we collect enough samples to document all or almost all of the plants consumed by northern Idaho ground squirrels?). We used Pianka's niche overlap index (Pianka, 1973) implemented in the spaa package in R (Zhang, 2016) with our presence/absence data to compare similarity in diet among age, sex, and seasonal groups. We compared northern Idaho ground squirrel summer diet composition to (a) all vegetation quadrats, and (b) MCP vegetation quadrats. By comparing plants eaten to plants available at these two different scales, we were able to more rigorously examine which plants were preferred food. We did not include any *Pinus* because we did not sample any species above 1.0m high, and thus, did not fully sample the trees in each vegetation quadrat (we did include *Pinus* when comparing fecal sample groups only). We compared summer (not spring) fecal samples with the data from our vegetation quadrats because we conducted our vegetation sampling during the same time period that summer fecal samples were collected. Hence, any differences in frequency of genera between summer fecal samples and vegetation quadrats can provide inferences regarding preference or avoidance of those genera.

#### 2.9.2 Genus level evaluation of the diet data

We used the randomForest package in R (Liaw & Wiener, 2002, R Core Team 2017) to determine which plant genera best discriminated whether a sample came from: (a) a squirrel fecal sample versus a vegetation quadrat (based on all 383 vegetation quadrats), (b) a squirrel fecal sample versus a vegetation quadrat (based on only the 191 MCP vegetation quadrats), (c) an adult fecal sample collected in the summer versus a juvenile fecal sample collected in the summer, and (d) an adult female fecal sample versus and adult male fecal sample. Random forests are bagged decision tree models whereby multiple decision trees are built and then merged together to get a more accurate prediction (Liaw & Wiener, 2002). We used random forest models because they do not overfit the data and have high predictive accuracy (Breiman, 2001). Furthermore, random forests provide estimates of variable importance. Thus, we were able to identify which plants were best able to differentiate between groups (i.e., adult vs. juvenile, males vs. females, used vs. available). We tuned the random forest models to determine the number of variables (*n*) to try at each node of the tree, and the number of trees (*m*) to grow that resulted in the lowest out‐of‐bag (OOB) error rates. A random forest uses a random subset of predictive variables in the division of each node, which reduces the generalization error. Random forests also use bagging or bootstrap aggregating to make the trees grow from different training data subsets. The samples that are not present in the training subset are included as part of the out‐of‐bag (OOB). These OOB elements can be classified by the tree to evaluate the performance (accuracy). The proportion between the misclassification and the total number of OOB elements provides the unbiased estimation of error. We conducted all statistical analyses in R version 3.43 (R Core Team 2017). Furthermore, we examined partial plots to identify whether plant taxa used to discriminate between groups were more common in one group or the other.

#### 2.9.3 Apparent overwinter survival

We used a generalized logistic regression model implemented in program R version 3.43 (glm model with a binomial family and a logit‐link) to assess whether the presence of individual plant genera in the summer diet impacted apparent overwinter survival of northern Idaho ground squirrels (*n* = 124; 53 adults and 71 juveniles). We assigned a squirrel as “survived” if it was retrapped in any year following the summer that its fecal sample was collected whereas we assumed a squirrel had “not survived” if it was never retrapped again in subsequent years. We used the term apparent overwinter survival because we cannot distinguish between a squirrel that truly died over the winter and one that dispersed away from the trapping area between the summer trapping season and the following spring trapping season. However, we believe the probability of recapture of those within the trapping area (in the unlikelihood we failed to recapture an individual who was still alive and within our trapping area) is likely equal between those that consumed and did not consume each plant. We included the 13 plant genera that were detected in ≥25% of the summer northern Idaho ground squirrel pellet samples because we were interested in addressing how each of the 13 most common plant genera in the diet affected squirrel survival. We used Akaike's Information Criterion corrected for small sample size (AIC_c_) to compare a suite of candidate models (Akaike, 1974). All survival analyses were implemented in program R version 3.6.2. All models also included squirrel age as a predictor variable because overwinter survival of juveniles is lower than adults (Sherman & Runge 2002). We ran a separate model for each of the genera to evaluate whether age should be included as an additive or interaction. Inclusion of an interaction between age and presence–absence of the plant did not significantly improve the models (*p* > .05 for all 13 models), so we did not include interaction terms.

All top models included age as an additive and hence we only ran fully additive models. We used package MuMIn (Barton, 2020) in program R to evaluate all additive combinations of the 13 genera and sex. Each plant was modeled as present or absent (binary predictor variable).

## 3 RESULTS

**Figure 3:**
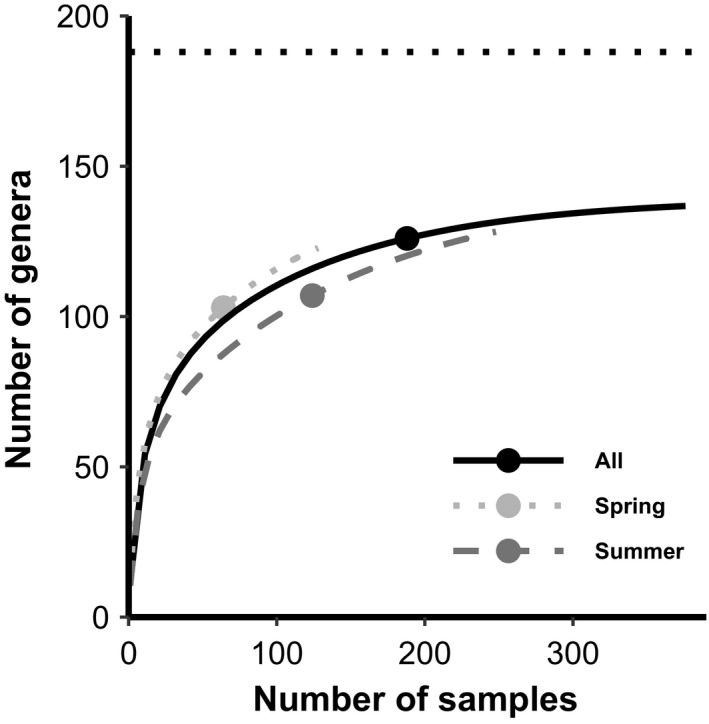
Sample‐based rarefaction curves for spring only (64), summer only (124), and all fecal samples combined (188). We extrapolated the curves past the points (number of samples) to double the number of samples in each category to better illustrate the projection of the accumulation curve. The three points (circles) show the number of genera actually detected for each category. The horizontal dotted line at 188 genera represents the 188 genera that were identified at our field sites (a combination of genera we identified during our vegetation sampling and genera identified by previous studies at the same field sites) and represent the likely maximum number of genera available to northern Idaho ground squirrels diets

We used genetic data informed by vegetation assessment to identify 42 families, 126 genera, and 120 species of plants (Table S1) in the 188 northern Idaho ground squirrel fecal samples. Each of the three different gene regions identified a similar number of total genera within the northern Idaho ground squirrel diet (Table S2). However, 6.3% (ITS2), 10.9% (ITS1), and 18.0% (*trnL*) of the genera were only picked up by one of three gene regions. Thus, we would have identified only 83–89 genera if we had used only one gene region. The ITS1 region identified the greatest number of genera (89) (six more than ITS2 and five more than *trnL*). Every fecal sample (100%) contained at least one species of forbs, and 85.1% of the fecal samples contained at least one species of grass. Trees occurred in 32.4% of the samples, and shrubs occurred in 12.2% of the samples. Rushes and sedges were rarely detected in fecal samples (4.3%). Adult spring fecal samples contained 80.2% more shrubs and 53.5% more trees than summer fecal samples. In contrast, adult summer fecal samples contained 13.8% more grasses and 18.9% more rushes and sedges than spring fecal samples. The frequency of fecal samples containing forbs, grasses, shrubs, trees, and rushes/sedges significantly differed between spring and summer (*p* = .044, two‐tailed Fisher's exact test). Rarefaction curves based on the 124 summer samples came close to approaching the asymptote, indicating that our sample size of fecal samples was effective at documenting the diverse diet of squirrels (all seasons combined; Figure 3). However, more samples in the spring season were likely needed to more completely document the wide diversity of plants eaten during springtime (Figure 3).

On average, 11.36 (± 0.42 SE) plant genera were identified in each sample (range 1–31 genera per sample). On average, diets of adult ground squirrels contained more genera in the spring (13.19 ± 0.79 SE genera) than summer (9.47 ± 0.75 SE genera; *t* = 3.42, *df* = 113.8, *p* < 0.001). Adult northern Idaho ground squirrel fecal samples averaged 1.7 fewer genera than did juvenile fecal samples (9.47 ± 0.75 vs. 11.13 ± 0.62 *SE*) in the summer, but that difference was not significant (*t* = −1.70, *df* = 109.2, *p* = .09) and diet overlap was high between adults and juveniles (Table 2). Hence, diets of adults differed seasonally (spring vs. summer) more so than diets differed between age classes (juveniles vs. adults; Table 2).

**Table 2:** Dietary overlap in northern Idaho ground squirrels based on Pianka's niche overlap indices

	Spring			Summer							
	Adult Males	Adult Females	Adult	Adult Males	Juv Males	Males	Adult Females	Juv Females	Females	Adult	Juv
Spring Adult Females	0.888										
Spring Adults	0.956	0.984									
Summer Adult Males	0.718	0.766	0.768								
Summer Juv Males	0.788	0.810	0.824	0.895							
Summer Males	0.778	0.812	0.821	0.965	0.980						
Summer Adult Females	0.718	0.764	0.767	0.890	0.861	0.897					
Summer Juv Females	0.732	0.768	0.775	0.911	0.908	0.934	0.933				
Summer Females	0.738	0.779	0.785	0.918	0.904	0.934	0.975	0.989			
Summer Adults	0.738	0.786	0.789	0.963	0.900	0.952	0.980	0.949	0.977		
Summer Juv	0.771	0.803	0.812	0.925	0.965	0.974	0.925	0.986	0.978	0.951	
Summer All	0.766	0.805	0.812	0.952	0.950	0.976	0.959	0.983	0.989	0.983	0.992

Notes

Overlap values closer to 1.0 indicate more similar diets and overlap values closer to 0.0 indicate less overlap. We compared diets by season (spring and summer), sex (males and females), and age (adult and Juv: juvenile) categories.

**Figure 4:**
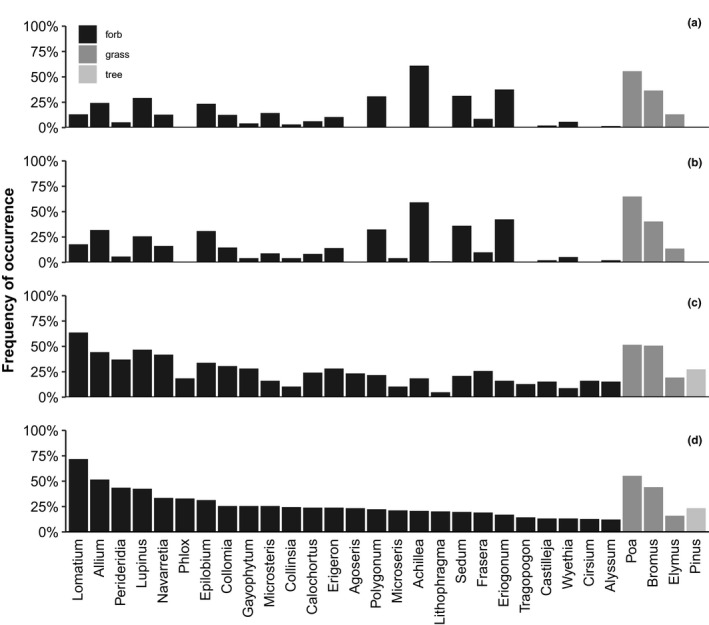
Frequency of occurrence of the 30 most‐common plant genera in a) all vegetation quadrats, b) MCP vegetation quadrats, c) summer fecal samples from northern Idaho ground squirrels, and d) all fecal samples (spring and summer samples) from northern Idaho ground squirrels. We included the summer fecal samples separately (panel c) because we only sampled the vegetation in the summer. We did not include *Pinus* in the vegetation quadrats (a, b) because we only sampled below 1‐m (we did not sample the upper canopy)

**Figure 5:**
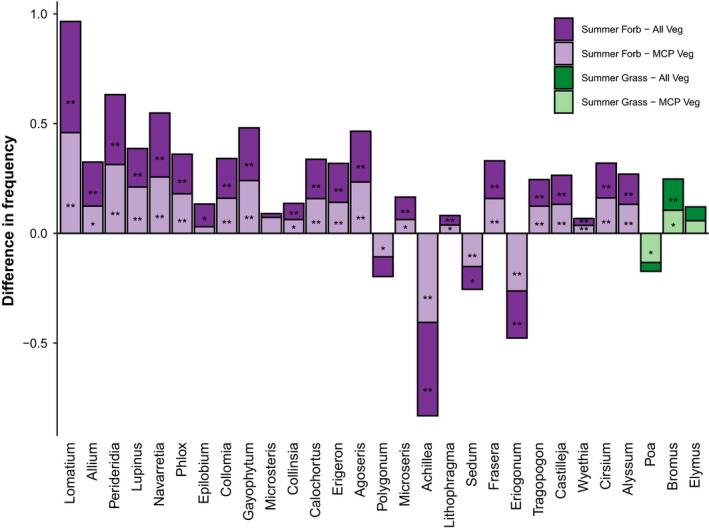
Difference in frequency between genera in summer fecal samples compared to all vegetation quadrats and summer fecal samples compared to MCP vegetation quadrats. Bars less than zero represent genera that were found more frequently in the vegetation quadrats (environment) compared to the fecal samples (diet) (i.e., those that squirrels may have avoided). We did not include *Pinus* because they were not assessed in the vegetation quadrats. Forb genera are in shades of purple and grass genera are shades of green. We used chi‐square tests to determine whether there was a significant difference between percent of each genera within fecal samples versus percent of each genera within either all vegetation quadrats or MCP vegetation quadrats (* < 0.05 and ** < 0.01)

*Lomatium, Poa*, and *Allium* were the three most‐common genera in the squirrel's diet (in >50% of the 188 fecal samples; Figure 4). Twenty‐four of the 29 most‐common plants identified in the northern Idaho ground squirrel diet were more frequently found in northern Idaho ground squirrel fecal pellets than in the vegetation quadrats (Figure 5). Only five genera were more common in the vegetation quadrats than in the northern Idaho ground squirrel fecal samples (Figure 5). Forty‐four genera were found in at least one vegetation quadrat and none of the fecal samples. The portion of the study sites frequently used by northern Idaho ground squirrels (i.e., the MCPs) were more likely to have the foods preferred by these squirrels (i.e., those more common in the pellets than expected; Figure S1) compared to the portion of the study sites outside of the MCPs. Only seven out of the 29 most common genera in the squirrel's diet were found more often in all vegetation quadrats than in the MCP vegetation quadrats (Figure S1).

**Figure 6:**
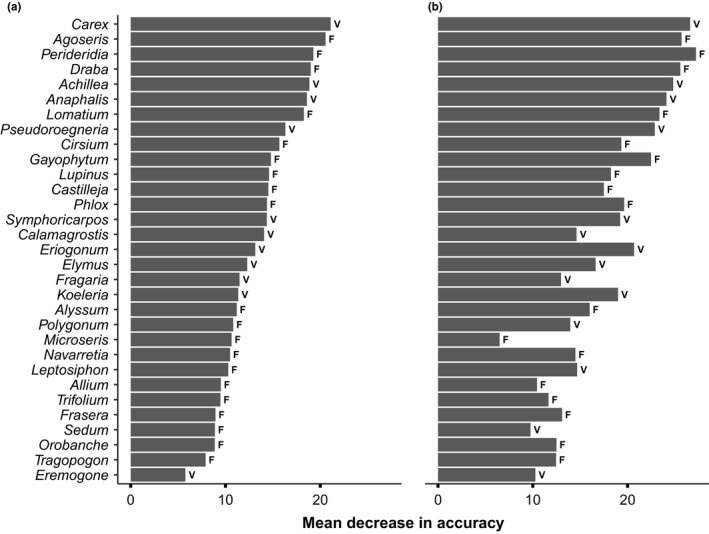
Variable importance contribution of: a) northern Idaho ground squirrel summer fecal samples versus 383 vegetation quadrats, and b) northern Idaho ground squirrel summer fecal samples versus a subset of 191 vegetation quadrats (those within northern Idaho ground squirrel MCPs at each study site). The mean decrease in accuracy is a measure of the impact of each plant genera on the accuracy of the model (e.g., if *Carex* were removed from the model in Panel a, the accuracy would be reduced by 21%). Only the top 30 genera are included in each panel representing the plant genera that are most important to the model's ability to distinguish between a sample from a fecal pellet and a sample from a vegetation quadrat. However, 31 are presented because *Eremogone* was only in the top 30 for the MCP vegetation quadrats but not for all quadrats and *Microseris* was only in the top 30 for all quadrats and not the MCP vegetation quadrats. A genus is more often found in a fecal sample if the letter “F” is next to the bar and is more often found in a vegetation quadrat if a “V” is next to the bar. No tree genera were included in this analysis because we did not sample trees in the vegetation quadrats. We did not include any trees because they were not included in the vegetation sampling (we only sampled vegetation below 1‐m height)

**Figure 7:**
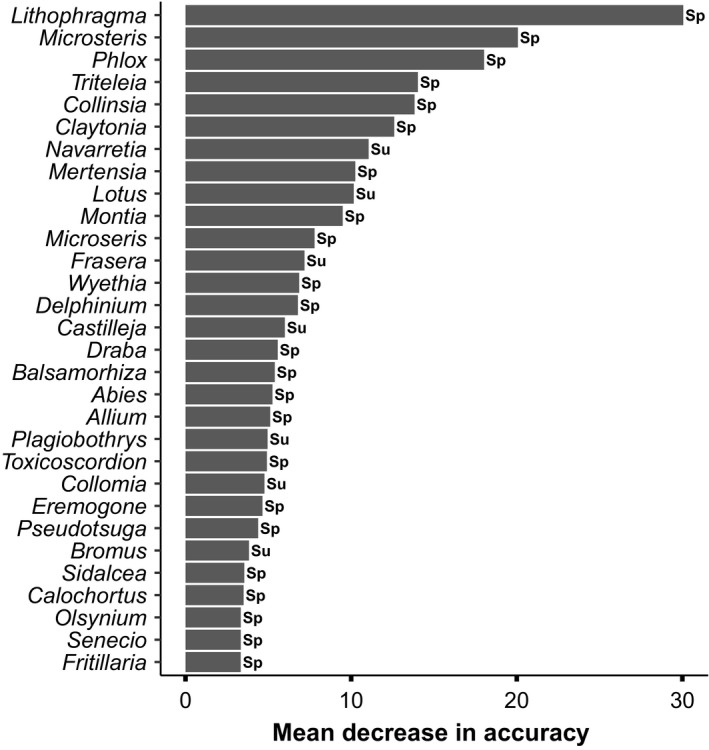
Variable importance contribution that shows which plant genera best discriminated between adult spring and summer fecal samples from northern Idaho ground squirrels. Letters next to each bar indicate whether a genus was more often found in spring diets (Sp) or summer diets (Su). Only the top 30 genera are included, representing the genera that are most important to the model's ability to distinguish between seasons

*Carex, Agoseris,* and *Perideridia* were the most important genera for discriminating between fecal samples and the vegetation quadrats (Figure 6). *Agoseris* and *Perideridia* were more often found in the summer fecal samples than in the vegetation quadrats, and *Carex* was more often found in the vegetation quadrats than fecal samples (Figure 6). *Agoseris* was found in 23.4% of the fecal samples and 0.0% of the MCP vegetation quadrats while *Perideridia* was found in 43.6% of the fecal samples and 6.3% of the MCP vegetation quadrats. *Carex* was found in only 3.2% of the fecal samples and 40.3% of the MCP vegetation quadrats. *Lithophragma, Microsteris*, and *Phlox* were the most important genera for predicting if a fecal sample was from the spring versus the summer (Figure 7). *Sidalcea, Calochortus*, and *Pinus* were the most important genera for predicting if a sample was from an adult versus a juvenile (Figure S2). However, differences between seasons (spring versus summer) were more pronounced than differences between age classes (adults versus juveniles), and the OOB (out‐of‐bag) error rate was relatively high (37.10%; Table S3), indicating that the models were better able to differentiate fecal pellets between seasons than those between age classes based on plant genera that they contained.

**Figure 8:**
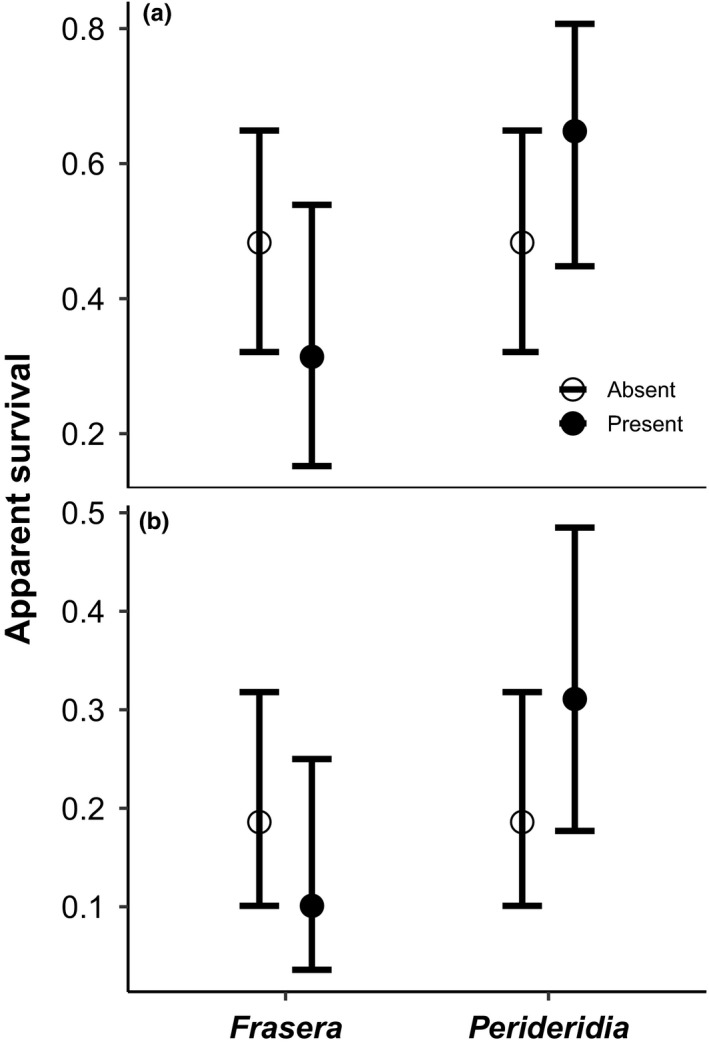
Difference in apparent overwinter survival for adult (a) and juvenile (b) northern Idaho ground squirrels based on whether a plant genus was present or absent in the squirrel's fecal sample. We sampled 124 individuals in the summer (71 juveniles and 53 adults). An animal survived if it was recaptured the following spring or any other subsequent trapping session. We assumed an animal died if it was never retrapped. Hence, we can only measure apparent survival because we cannot distinguish between those that dispersed off the trapping area versus those that actually died. Results are presented from the top competing model which included the additive effects of age, *Frasera spp*., and *Perideridia spp*. Vertical bars are 95% confidence intervals

Age was included in all the top survival models. The top model also contained *Frasera* and *Perideridia* (Table 3). There were 24 models with a delta AICc less than 2.0 suggesting model uncertainty. However, *Perideridia* was found in 66.7% of the top models and *Frasera* was found in 54.2% of the top models. Estimates from the top competing models suggest that overwinter survival was higher for individuals that consumed *Perideridia* versus those that did not (Figure 8). The top competing model (Age + *Frasera* + Perideridia) estimates were significant for Age (*p* < .001) but not for *Frasera* (*p* = .150) or *Perideridia* (*p* = .105). However, point estimates suggest that apparent overwinter survival was lower for individuals that consumed *Frasera* versus those that did not (Figure 8). Apparent overwinter survival was 1.34 and 1.67 times higher for individuals that consumed *Perideridia* compared to those that did not for adults and juveniles, respectively, and 1.54 and 1.84 times lower for those that consumed *Frasera* compared to those that did not for adults and juveniles, respectively.

**Table 3:** Top models that evaluated the relationship between overwinter survival and the 13 plant genera found in at least 25% of the northern Idaho ground squirrel fecal samples

	AIC_c_	ΔAIC_c_	*df*	w_i_
Age + Frasera +Perideridia	150.13	0.00	4	0.006
Age + Perideridia	150.19	0.05	3	0.006
Age + Allium +Frasera + Perideridia	150.33	0.20	5	0.006
Age + Frasera	150.64	0.51	3	0.005
Age + Allium +Frasera	150.73	0.59	4	0.005
Age	150.77	0.64	2	0.005
Age + Allium +Perideridia	151.39	1.26	4	0.003
Age + Naverretia +Perideridia	151.40	1.27	4	0.003
Age + Bromus +Perideridia	151.61	1.48	4	0.003
Age + Allium +Erigeron + Frasera +Perideridia	151.75	1.62	6	0.003
Age + Erigeron +Frasera + Perideridia	151.79	1.65	5	0.003
Age + Frasera +Lomatium + Perideridia	151.83	1.70	5	0.003
Age + Allium	151.84	1.71	3	0.003
Age + Erigeron +Perideridia	151.86	1.73	4	0.003
Age + Frasera +Poa	151.87	1.73	4	0.003
Age + Allium +Frasera + Poa	151.93	1.80	5	0.003
Age + Lomatium +Perideridia	151.94	1.81	4	0.003
Age + Gayophytum +Perideridia	151.96	1.83	4	0.002
Age + Bromus +Frasera + Perideridia	151.99	1.85	5	0.002
Age + Poa	152.00	1.86	3	0.002
Age + Allium +Frasera + Naverretia +Perideridia	152.03	1.90	6	0.002
Age + Frasera +Naverretia + Perideridia	152.04	1.91	5	0.002
Age + Naverretia	152.13	1.99	3	0.002
Age + Frasera +Perideridia + Poa	152.13	2.00	5	0.002
Null	159.42	9.29	1	0.000
Global	172.35	22.22	15	0.000

Notes

We also included Age in the model because previous published work showed that overwinter survival differs between juveniles and adults. We did not find any evidence that age had an interactive relationship with any of the 13 plant genera so we only evaluated additive models. We have only included the top competing models (those with a ΔAIC_c_ greater than or equal to 2.0) and both the null and global model for comparison

## 4 DISCUSSION

Our study is one of only a few studies that have documented diet of herbivores with resolution to the genus of forage plants; most other studies have done so at the family level (Iwanowicz et al., 2016). Furthermore, our study is the only DNA metabarcoding study that used three gene regions to document diet of an herbivore based on fecal samples and to document why using three gene regions (rather than one) provides a more complete and accurate description of an animal's diet breadth. Most previous studies focused on *trnL* alone or used a second region to gain resolution within only one or two families (Kartzinel et al., 2015; Lopes et al., 2015). Reliance on only one gene region (e.g., only *trnL*) would have caused us to miss >30% of the taxa in this rare squirrel's diet, including several of the most‐common forage plants. Consequently, the use of multiple gene regions is imperative for studies that use DNA metabarcoding methods to document diet of a generalist herbivore with a diverse diet. Our results demonstrate that DNA metabarcoding provides increased resolution compared to microhistology for quantifying the diet of herbivores and provides one of the few studies to document the extent to which DNA metabarcoding improves such resolution (Khanam, Howitt, Mushtaq, & Russell, 2016; Soininen et al., 2009). Furthermore, to our knowledge, this is the first DNA metabarcoding study to link specific forage plants to survival.

Our results corroborate and provide additional resolution to results from two prior microhistological studies: that the northern Idaho ground squirrel is a generalist herbivore with a diverse diet (Dyni & Yensen, 1996; Yensen et al., 2018). The diet of northern Idaho ground squirrels is dominated by forbs and grasses, with shrubs, trees, rushes, and sedges accounting for a much smaller component. However, we identified ~90 more plant species (300% increase) and ~70 more genera (125% increase) in the squirrels’ diet than previous microhistological studies (Dyni & Yensen, 1996; Yensen et al., 2018). Furthermore, three frequently occurring genera detected by DNA metabarcoding in the fecal samples (*Navarretia, Phlox*, and *Epilobium*) were not mentioned in one or both of two previous microhistological studies (Dyni & Yensen, 1996; Yensen et al., 2018). However, we did not detect two genera (*Descurainia* and *Amelanchier*) reported by Dyni and Yensen (1996) and six genera (*Cerastium, Hedysarum*, *Phleum*, *Equisetum*, *Pedicularis*, and *Saxifraga*) reported by Yensen et al. (2018) in our fecal samples. Both *Cerastium* and *Hedysarum* (of the eight genera we did not detect in our fecal samples) were only identified by Yensen et al. (2018) at one site where we did not sample. Five of the eight genera (*Cerastium, Hedysarum, Descurainia*, *Equisetum*, and *Pedicularis*) were not detected in any of our 383 vegetation quadrats at our 13 study sites. We found no records of *Hedysarum* in Adams County (CPNWH, 2018). Both *Pedicularis* and *Equisetum* are associated with wet areas (northern Idaho grounds squirrels live in drier areas). Hence, we are not surprised that we did not detect *Pedicularis* or *Equisetum* in either our fecal or vegetation samples. However, it is likely that squirrels moved in and out of our study sites and may have occasionally consumed vegetation that was adjacent and in wetter areas. Dyni and Yensen (1996) only reported plants found in over 1% of their samples, and this may partially explain why our list includes more plants. However, of the 78 genera that we detected that they did not detect, 82.1% were detected in >1% of our 188 fecal samples and 15.4% were detected in >10% of our 188 fecal samples. While some of the differences among the three studies may merely reflect that these squirrels have a diverse diet that differs among study sites, failures to detect species that were in many samples in one study but absent from another may indicate methodological bias. Furthermore, we may have missed some plant species in our quadrats that were indeed present within our sites because we only sampled the available vegetation in the summer, after some plants may have desiccated beyond our ability to identify them.

Northern Idaho ground squirrels consumed some plant genera (e.g., *Lomatium*) more than expected based on their frequency within the squirrels’ foraging areas and consumed other plants (e.g., *Poa, Achillea*) less than expected based on their frequency (Figure 5). Our results suggest that northern Idaho ground squirrels are preferentially eating several plants that are relatively uncommon (e.g., *Phlox* and *Periderida*), a pattern that corroborates a previous diet study (Yensen et al., 2013), but they also eat some common plants (e.g., *Poa*). The protein content of most grasses decreases as the growing season progresses (Frase & Armitage, 1989), but squirrels frequently eat grass caryopses later in the summer when the leaves and stems of most herbaceous vegetation have senesced. Grasses invest less in chemical defenses than other plants (Crawley, 1997), but do contain more silica‐rich phytoliths than herbaceous vegetation. Grasses may also have less nutrients than forbs (Bennett, 1999); some herbivores select a diet containing both forbs and grasses to maximize energy intake and digestibility (Belovsky 1984, 1986). Optimal foraging theory predicts that when food is abundant, individuals are more likely to be choosy, and should select higher‐quality foods (Pyke, Pulliam, & Charnov, 1977). Small‐bodied hind‐gut fermenters, such as the northern Idaho ground squirrel, rely on low fiber and higher energy foods (Hume, Morgan, & Kenagy, 1993). Thus, smaller‐bodied sciurids typically consume less grass than larger‐bodied sciurids (Yensen et al., 2018). Perhaps squirrels are able to select forbs they prefer in the spring, but must incorporate less nutritious (but common) grasses as the summer progresses, to ensure they obtain sufficient energy to survive overwinter hibernation. Or, perhaps squirrels switch to eating grass caryopses in the summer because they provide essential fatty acids valuable for hibernation. To better understand seasonal changes in diet and forage preferences, we need more detailed information on nutrient content of different plant growth forms so that we can better understand how changes in environmental conditions will impact the interaction between herbivores and their forage. Habitat use of northern Idaho ground squirrels may reflect diet constraints; the 29 most commonly eaten plants were more common in squirrel use areas (MCPs) compared to the areas immediately surrounding the MCPs (Figure S1).

Northern Idaho ground squirrels spend the majority of their lifetime in hibernation, and we found some intriguing relationships between apparent overwinter survival and diet. Squirrels that consumed *Perideridia* tended to have higher survival, whereas squirrels that consumed *Frasera* tended to have lower survival. All parts of *Perideridia* are edible, and it was a primary food crop of indigenous peoples in western North America (Turner, 1981). *Perideridia* produces large tuberous roots underground (Clarke, 1977). *Perideridia* is relatively high in protein and energy content, and low in fiber, compared to many other plants (Eshelman & Jenkins, 1989). Furthermore, *Perideridia* is high in starch, vitamin A, vitamin C, and potassium (Kaldy, Johnston, & Wilson, 1980). In late June and July, after the plants have dried, squirrels are most likely consuming old stems, roots, tubers, or seeds. A recent microhistological study also reported that this species consumed underground parts of forbs in mid‐summer (Yensen et al., 2018), which corresponds with the timing of our sampling. As above‐ground vegetation dries during the summer, fiber typically increases, reducing digestibility (Elliott & Flinders, 1984). Future studies should compare the nutritional content of *Perideridia* and *Frasera* to help understand why they affect squirrel survival. The availability of *Perideridia* may influence the local distribution of northern Idaho ground squirrels; a hypothesis that would explain our results and deserves deductive testing.

Our analyses indicated that northern Idaho ground squirrel diet differed between spring and summer, and these results corroborate previous studies that have reported seasonal differences in the diet of northern Idaho ground squirrels and other rodents (Schitoskey Jr and Woodmansee, 1978, Fagerstone, Tietjen, & Williams, 1981; Frase & Armitage, 1989; Lehmer et al., 2006; Yensen et al., 2018). Different plants are available at different times throughout the growing season, and squirrels may be forced to alter their diet based on plant phenology. Adult northern Idaho ground squirrels consumed a greater number of plant genera in the spring than in the summer, and this pattern could reflect either seasonal changes in plant availability (functional response), seasonal changes in the squirrels’ nutritional requirements, or both. Future studies should sample vegetation (availability of different forage plants) during both spring and summer to help evaluate these two potential causes of the seasonal changes we recorded. One limitation of DNA metabarcoding (relative to microhistology) is the inability to determine which part of a plant (seeds vs. roots vs. leaves) the animal eats and, hence, whether the use of different plant parts varies seasonally. Many squirrel species shift to other plant parts (e.g., roots and seeds) based on availability (Fagerstone et al., 1981; Karasov, 1982). However, the seasonal shifts in plant parts consumed can potentially be inferred based on phenological data from herbarium specimens (Consortium of Pacific Northwest Herbaria Specimen Database(CPNWH), 2018). For example, northern Idaho ground squirrels consumed *Lithophragma* more often in the spring than in the summer (Figures 3 & 7). At the elevational range in which northern Idaho ground squirrels are found, *Lithophragma* goes to seed on the 9th of June (on average) within Adams County. This suggests that squirrels are targeting the leaves and flowers of *Lithophragma* during the spring (when seeds are not yet available). In contrast, *Lomatium* is commonly consumed both in the spring and summer (Figure 4), and it begins to grow early in the spring (Ogle & Brazee, 2009) and typically begins fruiting ~22 June. Hence, northern Idaho ground squirrels are most likely eating all parts (leaves, roots, flowers, and seeds) of *Lomatium*. We often observed signs of ground squirrels digging in the summer months, presumably to eat plant roots (such as *Lomatium* roots). Polyunsaturated fatty acids, particularly linoleic and linolenic acid, are critical nutrients for some hibernating mammals (Florant, 1998; Frank, Dierenfeld, & Storey, 1998; Munro & Thomas, 2004; Ruf & Arnold, 2008), and some plant seeds contain high levels of polyunsaturated fatty acids (Frank et al., 1998; Lehmer & Horne, 2001). Piute ground squirrels (*Urocitellus mollis*) and arctic ground squirrels (*Urocitellus parryii*) consume more shrubs in the summer than spring (McLean, 1985; Van Horne et al., 1998), and shrubs may be higher in linoleic acid (Van Horne et al., 1998). Northern Idaho ground squirrels may shift to seeds in late summer for the same reason.

Many species face a changing landscape (e.g., climate change or changes in land‐use), and it is imperative that we understand the relationship between diet and food availability to better inform future conservation efforts to specifically target the nutritional needs and forage preferences of rare species. We have demonstrated that new metabarcoding techniques offer a good alternative to microhistological techniques at evaluating the diet of herbivorous species. DNA metabarcoding techniques enable us to evaluate dietary differences at a lower taxonomic level which can be followed up to ascertain why species are selecting specific species of plants (i.e., are animals targeting specific nutrients, water, etc.). Furthermore, it is important to evaluate how diets change by season as both availability and need may differ and hence plant management goals may need to target different plants during different times of year. Thus, it is imperative that we understand why animals are selecting certain plants in the landscape because certain plants that were once common may be affected by anthropogenic changes. However, managers may be able to better target restoration efforts on nutrients rather than specific species of plants that can cope with a changing climate and hopefully generalist mammalian herbivores may be able to respond positively to these management actions.

## 5 CONCLUSION

Northern Idaho ground squirrels have a diverse diet that included >126 plant genera and differed seasonally. However, squirrels are selective in their foraging and their choices have consequences. Squirrels who consumed *Perideridia* prior to hibernation had higher survival than those that did not. Moreover, the results of DNA metabarcoding from fecal pellets were affected by the gene regions used; our use of three gene regions substantially increased our ability to identify the full diet breadth of ground squirrels. The ability to identify the entire dietary breadth of a rare herbivore greatly improves our ability to manage the landscape where they live.

## CONFLICT OF INTEREST

The authors declare no conflict of interest.

## AUTHOR CONTRIBUTION

**Amanda Goldberg:** Conceptualization (equal); Data curation (lead); Formal analysis (lead); Methodology (equal); Project administration (lead); Visualization (lead); Writing‐original draft (lead); Writing‐review & editing (lead). **Courtney J Conway:** Conceptualization (equal); Formal analysis (equal); Funding acquisition (lead); Methodology (equal); Project administration (equal); Writing‐original draft (equal); Writing‐review & editing (equal). **David C Tank:** Conceptualization (equal); Data curation (equal); Formal analysis (equal); Methodology (equal); Writing‐original draft (supporting); Writing‐review & editing (supporting). **Kimberly Andrews:** Formal analysis (equal); Methodology (equal); Writing‐original draft (supporting); Writing‐review & editing (supporting). **Lisette Waits:** Funding acquisition (equal); Methodology (supporting); Writing‐original draft (supporting); Writing‐review & editing (supporting). **Digpal Singh Gour:** Investigation (supporting); Methodology (supporting); Writing‐review & editing (supporting).

## Supporting information

Fig S1

Fig S2

Tables S1–S3

## Data Availability

The data and R code that support the findings of this study have been made available online through Dryad (https://doi.org/10.5061/dryad.fttdz08q9). Any requests that include or relate to specific locations of data should be made via email and will be considered and accommodated where appropriate given that the focal species is listed as federally threatened under the Endangered Species Act. Submit any such requests to: cconway@uidaho.edu.
