# Supplementary figures and images for: Diet of a rare herbivore based on DNA metabarcoding of feces: Selection, seasonality, and survival
Source: Ecol Evol. 2020 Jun 30;10(14):7627–43. doi: 10.1002/ece3.6488 (PMC7391308; doi:10.1002/ece3.6488)

Difference in Frequency

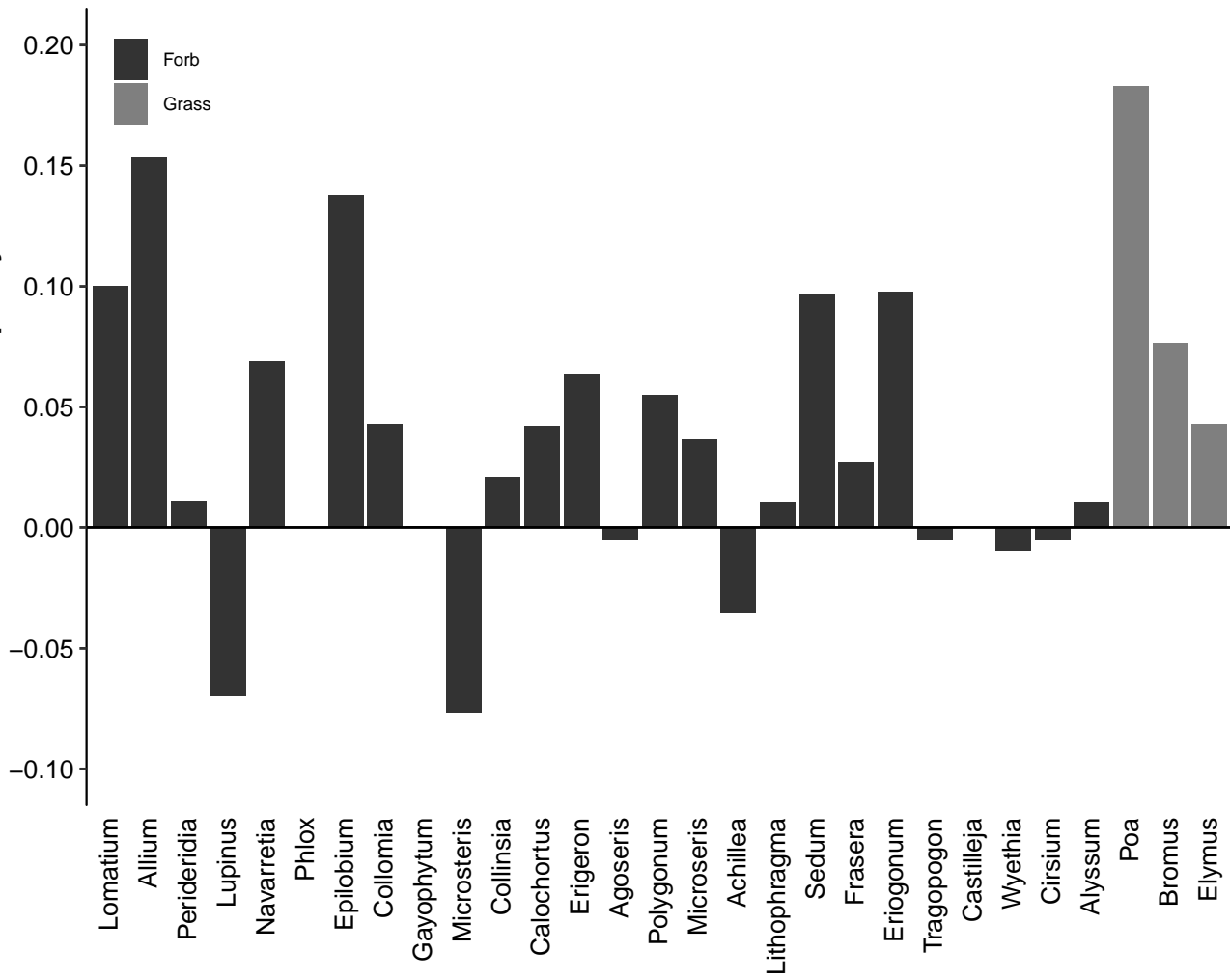

Supplement: Supplementary file 1 — Fig S1 [file ECE3-10-7627-s001.pdf]

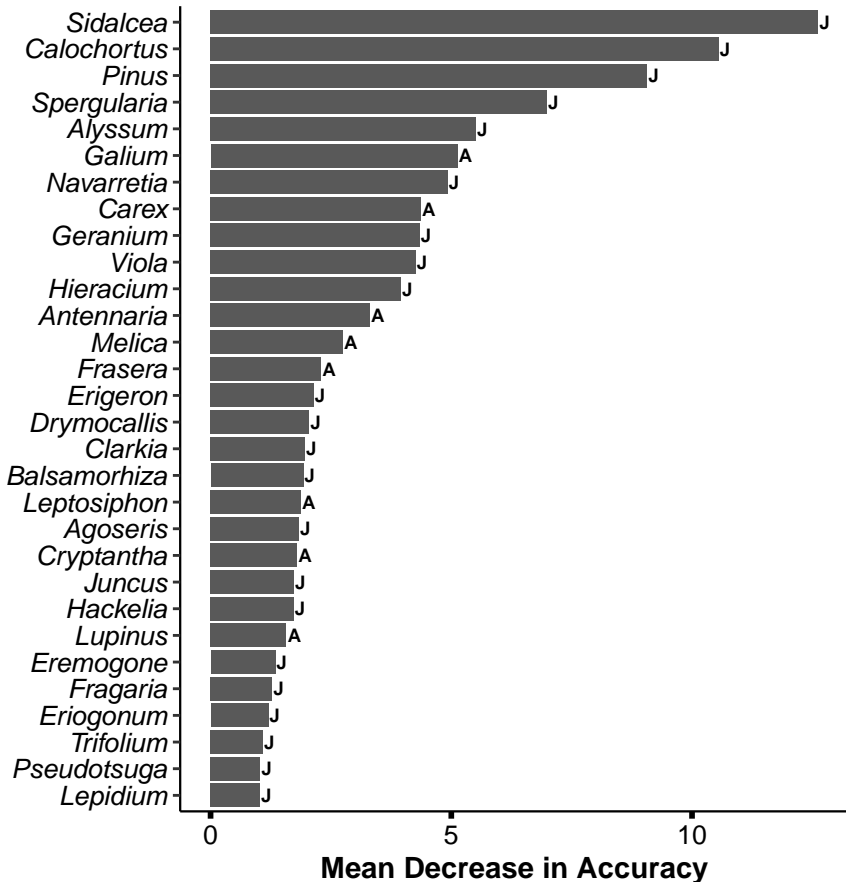

Supplement: Supplementary file 2 — Fig S2 [file ECE3-10-7627-s002.pdf]
