# Supplementary material for: Diet of a rare herbivore based on DNA metabarcoding of feces: Selection, seasonality, and survival
Source: Ecol Evol. 2020 Jun 30;10(14):7627–43. doi: 10.1002/ece3.6488 (PMC7391308; doi:10.1002/ece3.6488)
Supplement: Supplementary file 3 — Tables S1–S3 [file ECE3-10-7627-s003.docx]

**Appendix**

Table S1. Complete list of plant species: 1) used in our master reference list (plants we identified in our vegetation quadrats at our 13 study sites or were identified as present at our sites from past studies), or 2) identified as present in at least 1 northern Idaho ground squirrel fecal sample.

| Family | Species: Reference List | Species: Fecal Samples |
| --- | --- | --- |
| Amaryllidaceae | *Allium* | *Allium* |
|  | *Allium acuminatum* | *Allium acuminatum* |
|  | *Allium brandegeei* |  |
|  | *Allium douglasii* |  |
|  | *Allium fibrillum* |  |
|  | *Allium tolmiei* | *Allium tolmiei* |
| Apiaceae | *Ligusticum canbyi* |  |
|  | *Lomatium* | *Lomatium* |
|  | *Lomatium grayi* | *Lomatium grayi* |
|  | *Lomatium leptocarpum* |  |
|  | *Lomatium nudicaule* | *Lomatium nudicaule* |
|  | *Lomatium triternatum* | *Lomatium triternatum* |
|  | *Orogenia* | *Orogenia* |
|  | *Perideridia bolanderi* | *Perideridia bolanderi* |
|  | *Perideridia gairdneri* | *Perideridia gairdneri* |
| Apocynaceae | *Apocynum androsaemifolium* |  |
| Asparagaceae | *Camassia quamash* | *Camassia quamash* |
|  | *Dichelostemma congestum* |  |
|  | *Maianthemum racemosum* |  |
|  | *Maianthemum stellatum* |  |
|  | *Triteleia grandiflora* | *Triteleia grandiflora* |
| Asteraceae | *Achillea* |  |
|  | *Achillea millefolium* | *Achillea millefolium* |
|  | *Adenocaulon bicolor* |  |
|  | *Agoseris* | *Agoseris* |
|  | *Agoseris glauca* |  |
|  | *Agoseris heterophylla* | *Agoseris heterophylla* |
|  | *Anaphalis margaritacea* |  |
|  | *Antennaria* | *Antennaria* |
|  | *Antennaria luzuloides* |  |
|  | *Antennaria microphylla* | *Antennaria microphylla* |
|  | *Antennaria rosea* |  |
|  | *Arnica* | *Arnica* |
|  | *Arnica chamissonis* |  |
|  | *Arnica cordifolia* | *Arnica cordifolia* |
|  | *Arnica sororia* | *Arnica sororia* |
|  | *Artemisia* | *Artemisia* |
|  | *Artemisia arbuscula* |  |
|  | *Artemisia rigida* |  |
|  | *Artemisia tridentata* |  |
|  | *Artemisia tripartita* |  |
|  | *Aster* |  |
|  | *Balsamorhiza* | *Balsamorhiza* |
|  | *Balsamorhiza hookeri* |  |
|  | *Balsamorhiza sagittata* | *Balsamorhiza sagittata* |
|  | *Chrysothamnus viscidiflorus* |  |
|  | *Cirsium* | *Cirsium* |
|  | *Cirsium vulgare* |  |
|  | *Crepis* | *Crepis* |
|  | *Crepis acuminata* |  |
|  | *Crepis occidentalis* |  |
|  | *Ericameria nauseosus* |  |
|  | *Erigeron* | *Erigeron* |
|  | *Erigeron filifolius* | *Erigeron filifolius* |
|  | *Erigeron pumilus* | *Erigeron pumilus* |
|  | *Erigeron speciosus* |  |
|  | *Eriophyllum lanatum* |  |
|  | *Eurybia integrifolia* |  |
|  | *Gnaphalium* | *Gnaphalium* |
|  | *Grindelia hirsutula* |  |
|  | *Grindelia nana* | *Grindelia nana* |
|  | *Helianthella uniflora* | *Helianthella uniflora* |
|  | *Hieracium* | *Hieracium* |
|  | *Hieracium albiflorum* |  |
|  | *Hieracium scouleri* |  |
|  | *Hieracium triste* |  |
|  | *Madia* | *Madia* |
|  | *Madia glomerata* |  |
|  | *Madia gracilis* | *Madia gracilis* |
|  | *Microseris* |  |
|  | *Microseris nutans* | *Microseris nutans* |
|  | *Nothocalais nigrescens* | *Nothocalais nigrescens* |
|  | *Oreostemma alpigenum* |  |
|  | *Pyrrocoma* |  |
|  | *Pyrrocoma carthamoides* |  |
|  | *Senecio* | *Senecio* |
|  | *Solidago* | *Solidago* |
|  | *Solidago lepida* |  |
|  | *Solidago missouriensis* | *Solidago missouriensis* |
|  | *Taraxacum* |  |
|  | *Taraxacum ceratophorum* |  |
|  | *Taraxacum officinale* | *Taraxacum officinale* |
|  | *Tragopogon* | *Tragopogon* |
|  | *Tragopogon dubius* | *Tragopogon dubius* |
|  | *Wyethia* | *Wyethia* |
|  | *Wyethia amplexicaulis* |  |
|  | *Wyethia helianthoides* | *Wyethia helianthoides* |
| Berberidaceae | *Mahonia repens* |  |
| Boraginaceae | *Cryptantha* | *Cryptantha* |
|  | *Cryptantha affinis* | *Cryptantha affinis* |
|  | *Cynoglossum officinale* | *Cynoglossum officinale* |
|  | *Hackelia* | *Hackelia* |
|  | *Hackelia deflexa* | *Hackelia deflexa* |
|  | *Lappula redowskii* |  |
|  | *Lithospermum ruderale* |  |
|  | *Mertensia longiflora* | *Mertensia longiflora* |
|  | *Myosotis* |  |
|  | *Myosotis micrantha* |  |
|  | *Plagiobothrys* | *Plagiobothrys* |
|  | *Plagiobothrys leptocladus* |  |
| Brassicaceae | *Alyssum* | *Alyssum* |
|  | *Alyssum alyssoides* | *Alyssum alyssoides* |
|  | *Arabis* |  |
|  | *Boechera holboellii* |  |
|  | *Capsella bursa-pastoris* |  |
|  | *Descurainia* |  |
|  | *Draba verna* | *Draba verna* |
|  | *Lepidium* | *Lepidium* |
|  | *Lepidium campestre* |  |
|  | *Lepidium graminifolium* |  |
|  | *Lepidium latifolium* |  |
|  | *Lepidium virginicum* | *Lepidium virginicum* |
| Caprifoliaceae | *Linnaea borealis* |  |
|  | *Lonicera utahensis* | *Lonicera utahensis* |
|  | *Symphoricarpos* | *Symphoricarpos* |
|  | *Symphoricarpos albus* |  |
|  | *Symphoricarpos oreophilus* |  |
|  | *Moehringia lateriflora* |  |
| Caryophyllaceae | *Arenaria* | *Arenaria* |
|  | *Dianthus armeria* | *Dianthus armeria* |
|  | *Eremogone* | *Eremogone* |
|  | *Eremogone aculeata* |  |
|  | *Eremogone congesta* | *Eremogone congesta* |
|  | *Holosteum umbellatum* |  |
|  | *Moehringia lateriflora* | *Moehringia lateriflora* |
|  | *Moehringia macrophylla* | *Moehringia macrophylla* |
|  | *Silene menziesii* | *Silene menziesii* |
|  | *Spergularia rubra* | *Spergularia rubra* |
| Celastraceae | *Paxistima myrsinites* | *Paxistima myrsinites* |
|  | *Sedum stenopetalum* | *Sedum stenopetalum* |
| Cyperaceae | *Carex* | *Carex* |
|  | *Carex geyeri* | *Carex geyeri* |
|  | *Carex hoodii* |  |
|  | *Carex pachystachya* |  |
|  | *Carex petasata* |  |
| Equisetaceae | *Equisetum* |  |
| Ericaceae | *Chimaphila umbellata* |  |
|  | *Hypopitys monotropa* |  |
|  | *Vaccinium* | *Vaccinium* |
|  | *Vaccinium membranaceum* |  |
| Fabaceae | *Acmispon americanus* |  |
|  | *Arachis* | *Arachis* |
|  | *Astragalus* | *Astragalus* |
|  | *Lathyrus* | *Lathyrus* |
|  | *Lathyrus lanszwertii* |  |
|  | *Lathyrus nevadensis* |  |
|  | *Lotus purshianus* |  |
|  | *Lotus unifoliolatus* | *Lotus unifoliolatus* |
|  | *Lupinus* | *Lupinus* |
|  | *Lupinus argenteus* |  |
|  | *Lupinus laxiflorus* |  |
|  | *Lupinus leucophyllus* |  |
|  | *Lupinus polyphyllus* | *Lupinus polyphyllus* |
|  | *Lupinus sericeus* |  |
|  | *Medicago* | *Medicago* |
|  | *Thermopsis montana* |  |
|  | *Trifolium* | *Trifolium* |
|  | *Trifolium eriocephalum* |  |
|  | *Trifolium macrocephalum* | *Trifolium macrocephalum* |
|  | *Trifolium pratense* |  |
|  | *Vicia* |  |
|  | *Vicia americana* | *Vicia americana* |
| Gentianaceae | *Frasera albicaulis* | *Frasera albicaulis* |
|  | *Frasera speciosa* | *Frasera speciosa* |
|  | *Erodium cicutarium* | *Erodium cicutarium* |
|  | *Geranium viscosissimum* | *Geranium viscosissimum* |
| Grossulariaceae | *Ribes cereum* | *Ribes cereum* |
| Hydrophyllaceae | *Hydrophyllum capitatum* | *Hydrophyllum capitatum* |
|  | *Nemophila breviflora* | *Nemophila breviflora* |
|  | *Nemophila pedunculata* |  |
|  | *Phacelia* | *Phacelia* |
|  | *Phacelia franklinii* |  |
|  | *Phacelia hastata* |  |
| Hypericaceae | *Hypericum perforatum* |  |
|  | *Hypericum scouleri* |  |
| Iridaceae | *Olsynium douglasii* | *Olsynium douglasii* |
| Juncaceae | *Juncus* | *Juncus* |
|  | *Juncus parryi* |  |
| Lamiaceae |  |  |
|  | *Prunella vulgaris* |  |
|  | *Scutellaria angustifolia* |  |
| Liliaceae |  |  |
|  | *Calochortus* | *Calochortus* |
|  | *Calochortus elegans* | *Calochortus elegans* |
|  | *Calochortus eurycarpus* | *Calochortus eurycarpus* |
|  | *Erythronium grandiflorum* |  |
|  | *Fritillaria pudica* | *Fritillaria pudica* |
| Malvaceae | *Sidalcea oregana* | *Sidalcea oregana* |
|  | *Sidelacea* |  |
| Melanthiaceae | *Toxicoscordion* | *Toxicoscordion* |
|  | *Toxicoscordion paniculatum* |  |
|  | *Toxicoscordion venenosum* | *Toxicoscordion venenosum* |
|  | *Trillium ovatum* |  |
|  | *Veratrum* | *Veratrum* |
|  | *Veratrum californicum* |  |
|  | *Veratrum viride* |  |
| Montiaceae | *Claytonia* | *Claytonia* |
|  | *Claytonia cordifolia* |  |
|  | *Claytonia perfoliata* |  |
|  | *Claytonia rubra* | *Claytonia rubra* |
|  | *Montia linearis* | *Montia linearis* |
| Onagraceae | *Camissonia subacaulis* |  |
|  | *Chamerion angustifolia* |  |
|  | *Chamerion angustifolium* |  |
|  | *Clarkia pulchella* |  |
|  | *Clarkia rhomboidea* | *Clarkia rhomboidea* |
|  | *Epilobium* | *Epilobium* |
|  | *Epilobium angustifolium* |  |
|  | *Epilobium brachycarpum* | *Epilobium brachycarpum* |
|  | *Epilobium ciliatum* |  |
|  | *Gayophytum* | *Gayophytum* |
|  | *Gayophytum diffusum* | *Gayophytum diffusum* |
|  | *Calypso bulbosa* |  |
|  | *Goodyera oblongifolia* |  |
| Orobanchaceae | *Castilleja* | *Castilleja* |
|  | *Castilleja cusickii* |  |
|  | *Castilleja pallescens* |  |
|  | *Castilleja tenuis* |  |
|  | *Cordylanthus capitatus* | *Cordylanthus capitatus* |
|  | *Orobanche uniflora* | *Orobanche uniflora* |
|  | *Orthocarpus tenuifolius* | *Orthocarpus tenuifolius* |
|  | *Pedicularis* |  |
| Paeoniaceae | *Paeonia brownii* |  |
| Phrymaceae | *Mimulus breweri* |  |
|  | *Mimulus cusickii* |  |
| Pinaceae | *Abies* | *Abies* |
|  | *Abies concolor* |  |
|  | *Abies grandis* |  |
|  | *Pinus* | *Pinus* |
|  | *Pinus contorta* |  |
|  | *Pinus ponderosa* | *Pinus ponderosa* |
|  | *Pseudotsuga menziesii* | *Pseudotsuga menziesii* |
| Plantaginaceae | *Besseya rubra* |  |
|  | *Collinsia* | *Collinsia* |
|  | *Collinsia parviflora* | *Collinsia parviflora* |
|  | *Penstemon* | *Penstemon* |
|  | *Penstemon deustus* |  |
|  | *Penstemon gairdneri* |  |
|  | *Penstemon globosus* |  |
|  | *Penstemon wilcoxii* |  |
|  | *Plantago lanceolata* | *Plantago lanceolata* |
|  | *Veronica biloba* | *Veronica biloba* |
| Poaceae | *Achnatherum* |  |
|  | *Achnatherum nelsonii* | *Achnatherum nelsonii* |
|  | *Agropyron cristatum* |  |
|  | *Agropyron spicatum* |  |
|  | *Agrostis* | *Agrostis* |
|  | *Alopecurus pratensis* | *Alopecurus pratensis* |
|  | *Anthoxanthum* | *Anthoxanthum* |
|  | *Bromus* | *Bromus* |
|  | *Bromus arvensis* |  |
|  | *Bromus briziformis* |  |
|  | *Bromus carinatus* |  |
|  | *Bromus commutatus* | *Bromus commutatus* |
|  | *Bromus inermis* | *Bromus inermis* |
|  | *Bromus japonicus* | *Bromus japonicus* |
|  | *Bromus marginatus* |  |
|  | *Bromus rubens* |  |
|  | *Bromus tectorum* | *Bromus tectorum* |
|  | *Calamagrostis* |  |
|  | *Calamagrostis rubescens* | *Calamagrostis rubescens* |
|  | *Dactylis glomerata* | *Dactylis glomerata* |
|  | *Danthonia* | *Danthonia* |
|  | *Danthonia unispicata* |  |
|  | *Deschampsia* |  |
|  | *Deschampsia danthonioides* |  |
|  | *Deschampsia elongata* |  |
|  | *Elymus* | *Elymus* |
|  | *Elymus elymoides* |  |
|  | *Elymus multisetus* |  |
|  | *Festuca* | *Festuca* |
|  | *Festuca idahoensis* | *Festuca idahoensis* |
|  | *Festuca ovina* | *Festuca ovina* |
|  | *Festuca rubra* |  |
|  | *Koeleria macrantha* |  |
|  | *Leymus cinereus* |  |
|  | *Melica* | *Melica* |
|  | *Melica bulbosa* |  |
|  | *Melica spectabilis* |  |
|  | *Melica subulata* | *Melica subulata* |
|  | *Pascopyrum smithii* |  |
|  | *Phalaris arundinacea* | *Phalaris arundinacea* |
|  | *Phleum pratense* |  |
|  | *Poa* | *Poa* |
|  | *Poa bulbosa* | *Poa bulbosa* |
|  | *Poa compressa* | *Poa compressa* |
|  | *Poa nervosa* |  |
|  | *Poa pratensis* | *Poa pratensis* |
|  | *Poa secunda* | *Poa secunda* |
|  | *Pseudoroegneria spicata* | *Pseudoroegneria spicata* |
|  | *Thinopyrum intermedium* |  |
|  | *Ventenata dubia* |  |
| Polemoniaceae | *Collomia* | *Collomia* |
|  | *Collomia grandiflora* | *Collomia grandiflora* |
|  | *Collomia linearis* | *Collomia linearis* |
|  | *Ipomopsis aggregata* | *Ipomopsis aggregata* |
|  | *Leptosiphon harknessii* | *Leptosiphon harknessii* |
|  | *Microsteris gracilis* | *Microsteris gracilis* |
|  | *Navarretia* | *Navarretia* |
|  | *Navarretia capillaris* |  |
|  | *Navarretia divaricata* | *Navarretia divaricata* |
|  | *Navarretia intertexta* | *Navarretia intertexta* |
|  | *Phlox* | *Phlox* |
|  | *Phlox hoodii* |  |
| Polygonaceae | *Eriogonum* | *Eriogonum* |
|  | *Eriogonum douglasii* |  |
|  | *Eriogonum heracleoides* | *Eriogonum heracleoides* |
|  | *Eriogonum sphaerocephalum* |  |
|  | *Eriogonum umbellatum* | *Eriogonum umbellatum* |
|  | *Polygonum* | *Polygonum* |
|  | *Polygonum douglasii* | *Polygonum douglasii* |
|  | *Polygonum majus* | *Polygonum majus* |
|  | *Polygonum polygaloides* | *Polygonum polygaloides* |
|  | *Rumex* | *Rumex* |
|  | *Rumex acetosella* | *Rumex acetosella* |
|  | *Rumex utahensis* |  |
| Primulaceae | *Dodecatheon* |  |
|  | *Primula cusickiana* | *Primula cusickiana* |
| Ranunculaceae | *Anemone piperi* |  |
|  | *Clematis hirsutissima* | *Clematis hirsutissima* |
|  | *Delphinium* | *Delphinium* |
|  | *Delphinium bicolor* | *Delphinium bicolor* |
|  | *Ranunculus* | *Ranunculus* |
|  | *Ranunculus glaberrimus* |  |
|  | *Ranunculus uncinatus* |  |
|  | *Thalictrum occidentale* |  |
| Rhamnaceae | *Ceanothus* |  |
|  | *Ceanothus velutinus* | *Ceanothus velutinus* |
| Rosaceae | *Amelanchier alnifolia* |  |
|  | *Crataegus douglasii* | *Crataegus douglasii* |
|  | *Drymocallis* | *Drymocallis* |
|  | *Drymocallis arguta* |  |
|  | *Drymocallis glandulosa* | *Drymocallis glandulosa* |
|  | *Fragaria* | *Fragaria* |
|  | *Fragaria vesca* |  |
|  | *Fragaria virginiana* | *Fragaria virginiana* |
|  | *Geum triflorum* | *Geum triflorum* |
|  | *Physocarpus malvaceus* |  |
|  | *Potentilla* | *Potentilla* |
|  | *Potentilla gracilis* | *Potentilla gracilis* |
|  | *Potentilla recta* |  |
|  | *Poteridium annuum* | *Poteridium annuum* |
|  | *Prunus* | *Prunus* |
|  | *Prunus emarginata* |  |
|  | *Prunus virginiana* |  |
|  | *Purshia tridentata* | *Purshia tridentata* |
|  | *Rosa* | *Rosa* |
|  | *Rosa woodsii* |  |
|  | *Rubus parviflorus* |  |
|  | *Spiraea betulifolia* | *Spiraea betulifolia* |
| Rubiaceae | *Galium* | *Galium* |
|  | *Galium aparine* | *Galium aparine* |
|  | *Galium bifolium* |  |
|  | *Galium triflorum* |  |
|  | *Kelloggia galioides* |  |
| Salicaceae | *Populus tremuloides* |  |
|  | *Salix scouleriana* |  |
| Saxifragaceae | *Lithophragma* | *Lithophragma* |
|  | *Lithophragma glabrum* | *Lithophragma glabrum* |
|  | *Lithophragma parviflorum* | *Lithophragma parviflorum* |
|  | *Micranthes integrifolia* |  |
|  | *Saxifraga* |  |
| Scrophulariaceae | *Verbascum thapsus* |  |
| Violaceae | *Viola* | *Viola* |
|  | *Viola adunca* | *Viola adunca* |
|  | *Viola nuttallii* |  |
|  | *Viola purpurea* | *Viola purpurea* |

Table S2. Number of the 188 northern Idaho ground squirrel fecal samples that included each genera within the 3 gene regions sampled.

| Genus | ITS3_ITS4 | ITS5_ITS2 | *trnL* |
| --- | --- | --- | --- |
| *Abies* |  |  | 10 |
| *Achillea* | 2 | 38 | 2 |
| *Achnatherum* | 9 |  |  |
| *Agoseris* | 28 | 30 | 10 |
| *Agrostis* |  |  | 1 |
| *Allium* | 47 | 63 | 67 |
| *Alopecurus* |  |  | 1 |
| *Alyssum* | 14 | 17 | 20 |
| *Antennaria* | 1 |  | 2 |
| *Anthoxanthum* |  |  | 1 |
| *Arachis* |  |  | 1 |
| *Arenaria* |  | 1 |  |
| *Arnica* | 10 | 16 | 1 |
| *Artemisia* | 5 | 10 | 3 |
| *Astragalus* |  |  | 1 |
| *Balsamorhiza* | 2 | 18 |  |
| *Bromus* | 43 | 67 | 43 |
| *Calamagrostis* | 2 | 2 |  |
| *Calochortus* |  |  | 45 |
| *Camassia* |  | 1 | 2 |
| *Carex* | 2 | 3 | 2 |
| *Castilleja* | 1 | 6 | 22 |
| *Ceanothus* | 1 | 1 | 1 |
| *Cirsium* |  |  | 24 |
| *Clarkia* | 2 | 2 |  |
| *Claytonia* | 9 | 16 | 13 |
| *Clematis* |  | 3 | 2 |
| *Collinsia* | 20 | 38 | 24 |
| *Collomia* | 27 |  | 41 |
| *Cordylanthus* |  | 10 |  |
| *Coriandrum* |  |  | 1 |
| *Crataegus* |  |  | 1 |
| *Crepis* | 3 | 3 | 7 |
| *Cryptantha* | 2 | 6 | 3 |
| *Cynoglossum* | 1 | 2 |  |
| *Dactylis* |  | 2 |  |
| *Danthonia* | 1 |  |  |
| *Delphinium* |  |  | 9 |
| *Dianthus* |  | 1 |  |
| *Draba* | 5 | 4 | 6 |
| *Drymocallis* | 9 | 9 |  |
| *Elymus* | 12 | 28 |  |
| *Epilobium* | 11 | 45 | 29 |
| *Eremogone* | 3 | 6 | 2 |
| *Erigeron* | 33 | 36 |  |
| *Eriogonum* | 10 | 18 | 18 |
| *Erodium* |  |  | 1 |
| *Festuca* | 10 | 13 | 1 |
| *Fragaria* |  |  | 6 |
| *Frasera* | 23 | 29 | 29 |
| *Fritillaria* | 1 | 2 | 6 |
| *Galium* | 1 | 2 | 2 |
| *Gayophytum* | 34 | 22 | 22 |
| *Geranium* |  | 4 |  |
| *Geum* | 2 | 2 | 1 |
| *Gnaphalium* | 1 |  |  |
| *Grindelia* | 1 |  |  |
| *Hackelia* |  | 2 | 1 |
| *Helianthella* | 2 | 2 |  |
| *Hieracium* | 4 | 5 | 6 |
| *Hydrophyllum* |  |  | 2 |
| *Ipomopsis* | 1 |  | 1 |
| *Juncus* |  | 2 | 1 |
| *Lathyrus* |  |  | 1 |
| *Lepidium* | 1 | 1 | 1 |
| *Leptosiphon* | 5 | 4 |  |
| *Linanthus* |  | 1 |  |
| *Lithophragma* | 29 | 35 |  |
| *Lomatium* | 118 | 81 | 1 |
| *Lonicera* |  | 2 |  |
| *Lotus* | 5 | 9 | 13 |
| *Lupinus* | 41 | 58 | 65 |
| *Madia* | 20 |  | 5 |
| *Medicago* |  |  | 2 |
| *Melica* |  | 1 | 3 |
| *Mertensia* | 5 | 6 |  |
| *Microseris* | 21 | 33 |  |
| *Microsteris* |  | 57 |  |
| *Moehringia* |  |  | 2 |
| *Montia* | 6 | 5 | 6 |
| *Navarretia* | 18 | 58 | 22 |
| *Nemophila* | 3 |  |  |
| *Nothocalais* |  | 1 |  |
| *Olsynium* |  | 3 | 3 |
| *Orobanche* | 6 |  | 9 |
| *Orogenia* |  | 2 |  |
| *Orthocarpus* |  | 1 | 1 |
| *Paxistima* |  | 1 | 1 |
| *Penstemon* | 3 | 11 | 11 |
| *Perideridia* | 60 | 74 |  |
| *Phacelia* | 1 | 2 |  |
| *Phalaris* |  | 1 |  |
| *Phlox* | 46 |  | 58 |
| *Pinus* | 5 |  | 41 |
| *Plagiobothrys* | 10 | 11 |  |
| *Plantago* | 1 | 1 | 1 |
| *Poa* | 57 | 95 | 21 |
| *Polygonum* | 1 | 38 | 6 |
| *Potentilla* | 11 | 9 | 4 |
| *Poteridium* | 1 | 1 |  |
| *Primula* | 2 | 3 | 3 |
| *Prunus* | 1 | 1 | 1 |
| *Pseudoroegneria* | 1 |  |  |
| *Pseudotsuga* |  |  | 9 |
| *Purshia* |  | 2 |  |
| *Ranunculus* | 2 |  | 3 |
| *Ribes* | 1 | 1 | 1 |
| *Rosa* | 1 |  |  |
| *Rumex* |  | 2 |  |
| *Sedum* | 22 | 28 | 31 |
| *Senecio* | 5 | 9 |  |
| *Sidalcea* | 11 | 11 | 3 |
| *Silene* | 1 | 1 | 1 |
| *Solidago* | 3 | 5 |  |
| *Spergularia* | 6 | 7 |  |
| *Spiraea* |  |  | 2 |
| *Symphoricarpos* |  |  | 10 |
| *Taraxacum* | 7 | 5 |  |
| *Toxicoscordion* | 1 | 6 | 4 |
| *Tragopogon* | 25 | 3 |  |
| *Trifolium* | 14 | 19 | 20 |
| *Triteleia* |  |  | 18 |
| *Vaccinium* |  | 1 |  |
| *Veratrum* | 1 |  |  |
| *Veronica* |  |  | 2 |
| *Vicia* |  |  | 2 |
| *Viola* | 3 | 7 | 17 |
| *Wyethia* | 21 | 10 |  |
| **Total number of genera** | **83** | **89** | **84** |

Table S3. Optimal values for the random forest models used to classify 6 comparisons of fecal and/or vegetation samples.

| Model Comparison | Number of Variables at Each Split | Number of Trees | OOB Error Rate |
| --- | --- | --- | --- |
| All fecal samples vs all vegetation quadrats | 7 | 800 | 5.80% |
| All fecal samples vs MCP vegetation quadrats | 6 | 1000 | 5.30% |
| Summer fecal samples vs all vegetation quadrats | 12 | 600 | 3.15% |
| Summer fecal samples vs MCP vegetation quadrats | 7 | 1500 | 5.06% |
| Spring fecal samples vs summer fecal samples (adults only) | 12 | 1000 | 9.48% |
| Adult fecal samples vs juvenile fecal samples (summer only) | 8 | 800 | 37.10% |

Figure S1. Difference in frequency between genera in all northern Idaho ground squirrel MCP vegetation quadrats and all other quadrats (those outside of the MCPs). Bars less than zero represents genera that were more frequent outside the MCPs compared to within the MCPs. We did not include *Pinus* because they were not assessed in the vegetation quadrats.

Figure S2. Variable importance contribution that shows which plant genera best discriminate between adult and juvenile fecal samples from northern Idaho ground squirrels (*Urocitellus brunneus*) collected during the summer trapping session. Letters next to each bar indicate whether a genus was more often found in adult diets (A) or more often found in juvenile diets (J). Only the top 30 genera are included, representing the genera that are most important to the model’s ability to distinguish between age classes.
